# Dark-zone alterations expand throughout Paleolithic Lascaux Cave despite spatial heterogeneity of the cave microbiome

**DOI:** 10.1186/s40793-023-00488-8

**Published:** 2023-04-10

**Authors:** Zélia Bontemps, Claire Prigent-Combaret, Alice Guillmot, Mylène Hugoni, Yvan Moënne-Loccoz

**Affiliations:** 1 CNRS, INRAE, VetAgro Sup, UMR5557 Ecologie Microbienne, University of Lyon, Université Claude Bernard Lyon 1, 43 Bd du 11 Novembre 1918, 69622 Villeurbanne, France; 2 CNRS, INSA de Lyon, UMR Microbiologie Adaptation et Pathogénie, University of Lyon, Université Claude Bernard Lyon 1, 69622 Villeurbanne, France; 3 Institut Universitaire de France (IUF), Paris, France

**Keywords:** Lascaux Cave, Cave alteration, Decisional matrix, Anthropization, Unbalanced microbiota, Dark zones

## Abstract

**Background:**

Cave anthropization related to rock art tourism can lead to cave microbiota imbalance and microbial alterations threatening Paleolithic artwork, but the underpinning microbial changes are poorly understood. Caves can be microbiologically heterogeneous and certain rock wall alterations may develop in different rooms despite probable spatial heterogeneity of the cave microbiome, suggesting that a same surface alteration might involve a subset of cosmopolitan taxa widespread in each cave room. We tested this hypothesis in Lascaux, by comparing recent alterations (dark zones) and nearby unmarked surfaces in nine locations within the cave.

**Results:**

Illumina MiSeq metabarcoding of unmarked surfaces confirmed microbiome heterogeneity of the cave. Against this background, the microbial communities of unmarked and altered surfaces differed at each location. The use of a decision matrix showed that microbiota changes in relation to dark zone formation could differ according to location, but dark zones from different locations displayed microbial similarities. Thus, dark zones harbor bacterial and fungal taxa that are cosmopolitan at the scale of Lascaux, as well as dark zone-specific taxa present (i) at all locations in the cave (i.e. the six bacterial genera *Microbacterium, Actinophytocola, Lactobacillus*, *Bosea, Neochlamydia* and *Tsukamurella*) or (ii) only at particular locations within Lascaux. Scanning electron microscopy observations and most qPCR data evidenced microbial proliferation in dark zones.

**Conclusion:**

Findings point to the proliferation of different types of taxa in dark zones, i.e. Lascaux-cosmopolitan bacteria and fungi, dark zone-specific bacteria present at all locations, and dark zone-specific bacteria and fungi present at certain locations only. This probably explains why dark zones could form in various areas of the cave and suggests that the spread of these alterations might continue according to the area of distribution of key widespread taxa.

**Supplementary Information:**

The online version contains supplementary material available at 10.1186/s40793-023-00488-8.

## Background

Karst environments have attracted tourist interest for several centuries, especially when caves harbor Paleolithic art forms [1]. Rock art tourism has intensified in the last century and has significantly altered environmental conditions in several underground systems. The impact of tourism results from the initial opening of caves to the outside environment (initiation of air exchange) as well as the developments implemented to facilitate tourism, such as floor excavations (e.g. King Salomon Cave in Tasmania and Lascaux Cave in France), installation of air management machinery (operated from 1958 to early 2015 in Lascaux Cave), stairs, concrete paths, and artificial light [2–7]. Visitors themselves can also cause strong environmental imbalance in karst ecosystems, with an increase in temperature (about 0.20 °C in Tito Bustillo cave in Spain and 1.5 °C in Lascaux Cave compared with the original temperatures [3, 8];), CO_2_ concentrations and water vapor levels [4, 8–10]. As a result, the ecological disturbance suffered by show caves has impacted microbial structure, species richness and functioning [7, 11, 12].

Cave microbiota imbalance due to anthropization can cause microbial alteration of rock surfaces, which may threaten Paleolithic artwork. These alterations include (i) biodeterioration of the walls (implicating e.g. Actinobacteria and Ascomycota phyla) [13], (ii) lampenflora related to light systems, which is mainly composed of Cyanobacteria and Chlorophytes, and termed green disease in the 1960s in Lascaux [14–16], (iii) the development of calcite veil, possibly involving bacterial genera such as *Pseudomonas, Bacillus* and *Myxococcus* and masking art on the walls of the Large Cave of Arcy-sur-Cure in France [17], (iv) gypsum efflorescence [7] and (v) stains of different colors e.g. white, yellow and gray stains in Altamira Cave (Spain) [18, 19], red stains attributed to the outgrowth of bacteria (e.g. *Xanthomonodales, Thauera*, etc.) and fungi (e.g. *Exophiala, Acremonium*, etc.) [7, 18] in the Cave of Bats (Spain) [20], white stains caused early 2001 by *Fusarium solani* [5] and black stains (termed black disease) since the end of 2001 and attributed to black melanized fungi such as *Ochroconis* (= *Scolecobasidium*) *lascauxensis* [5] in Lascaux Cave. In several caves, stain mitigation was attempted by spraying antibiotics and chemical treatments (e.g. benzalkonium chloride) on cave walls, but this can accentuate further the imbalance of the cave microbiota as found in Lascaux [5, 6]. In the case of Lascaux Cave, the most recent type of surface alteration, termed dark zone, has developed in the Apse room over the past 15 years [21, 22].

Lascaux Cave is a model of particular interest, as it is arguably the most anthropized Paleolithic cave, and knowledge on surface alteration processes there can provide baseline information for the understanding and management of Paleolithic caves elsewhere. Among surface alterations, dark zones are the main threats currently as they keep growing, at the rate of a few cm each year. In comparison with unaltered surfaces, dark zones showed differences in the relative abundance of many genera, as *Ochroconis* fungi proliferate and *Pseudomonas* bacteria are counter-selected [21]. These changes are concomitant with the development of Bacteroidota and the bacterial genus *Labrys* (among others) from the onset of dark zone formation [22]. These findings point towards a sudden community switch in relation to dark zone formation, with rapid microbial successions leading to variations in microbial diversity and interaction networks [22].

Till recently, Lascaux’s dark zones were thought to be restricted to the Apse and adjacent walls in the Nave. However, close monitoring in several other rooms of the cave (e.g. farther in the Nave, Passage, Hall of Bulls, etc.) evidenced visual changes resembling those of the Apse’s dark zones on several rock walls (as well as on artificial limestone walls built to organize visits), leading to the hypothesis that similar microbiota changes (associated to dark zone formation) could be taking place in these different rooms. Yet, previous results showed that Lascaux’s microbiota was spatially heterogeneous when comparing different surfaces within a room [23] or a same type of surface in two different rooms [12], despite strong uniformity of climatic conditions. This probably applies at a wider scale within Lascaux (and other caves), and it raises the hypothesis that at least part of the changes in microbial community related to the formation of dark zones involve the same taxa throughout the cave, despite the microbiota specificities of the different rooms or surfaces.

The objective of this study was to assess microbiota particularities of cave wall alterations in heterogeneous caves, using the model case of dark zone alterations in Lascaux Cave. First, we compared microbial communities of unmarked limestone surfaces in nine areas of the cave. Second, we explored the microbial communities associated to dark zones in these nine areas, in comparison with the corresponding communities of unmarked surfaces nearby. Third, we assessed using a decisional matrix the level of similarity of the different dark zones relatively to the reference situation of the Apse, where dark zones appeared first and have already been characterized. Fourth, we investigated to which extent the formation of dark zones involved microbial proliferation, based on qPCR and (where permitted) scanning electron microscopy.

## Materials and methods

### Sample collection

Lascaux Cave is located near Montignac in Périgord, South-West France (N 45°03′13.087″ and E 1°10′12.362″). Touristic visits were stopped in 1963. Human presence is now highly restricted and restrained to scientific campaigns and official visits. The upper inclined plane at the left side of the Apse (hereafter termed ‘Apse’) was selected for reference sampling due to the first identification of a dark zone in June 2008 (based on photographic archives).

**Fig. 1:**
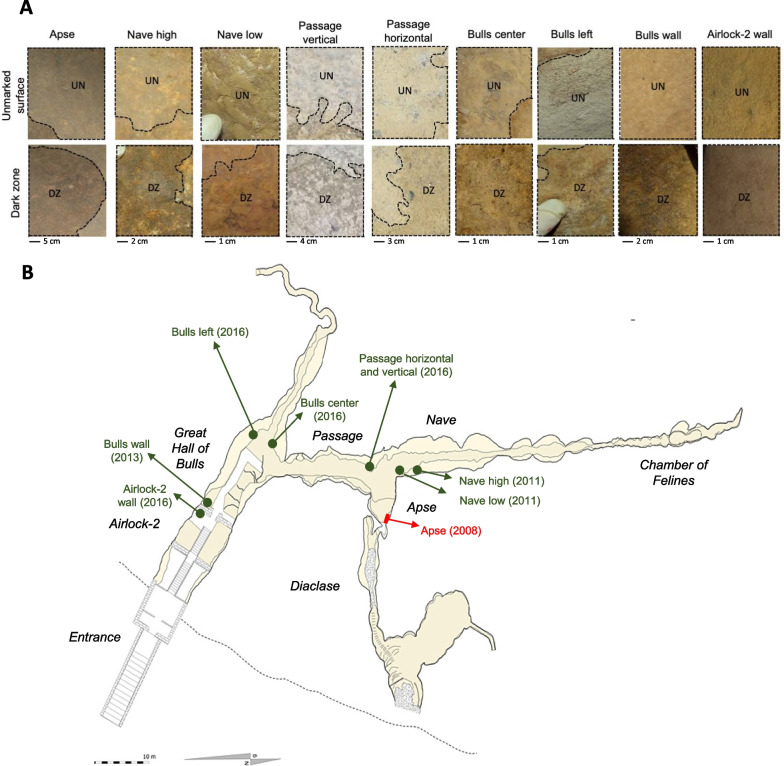
Photographs of dark zones sampled (**A**) and map of Lascaux Cave with the corresponding locations (**B**). In **A**, DZ means dark zone and UN means unmarked surface (source of the photographs: S. Géraud, DRAC Nouvelle Aquitaine). In **B**, the reference situation in the Apse in indicated in red, and the others in dark green (source of the map: S. Konik, Centre National de la Préhistoire). The year in which dark zones were first noticed is indicated in parenthesis

Other dark zones, similar to Apse’s dark zones, were sampled in eight other different areas (Fig. 1A), i.e. (i) two natural calcareous surfaces in the upper inclined planes (hereafter termed ‘Nave high’) and the low vertical wall (‘Nave low’) of the right side of the Nave (dark zones first noticed in July 2011 for both), (ii) four masonry benches made of local limestone particles and mortar, in the vertical part (‘Passage vertical’; dark zones first noticed in September 2016) and the horizontal part (‘Passage horizontal’; dark zones first noticed in March 2016) of the left bench (built in 1957 and modified in 1963) at the end of the Passage, and the vertical parts of the left bench (‘Bulls left’; built in 1957–1958) and central bench (‘Bulls center’; built in 1947–1948) at the back of the Hall of the Bulls (dark zones first noticed in April 2016 for both), and (iii) the two sides of the left stone wall (built in 1957–1958 using Santonian limestone blocks from a local quarry, with joints made of sand mixed with lime and/or cement) separating the Hall of the Bulls and the Airlock-2 entrance zone (‘Airlock-2 wall’ and ‘Bulls wall’; dark zones first noticed in October 2016 and June 2013, respectively) (Fig. 1B). All areas sampled had received chemical treatments in the past, but none since the formation of dark zones.

Sampling of the nine areas for molecular analyses was performed in September 2020, using swabs (synthetic sterile dry swab with cotton tip; Labomoderne, Gennevilliers, France) that were rubbed against about 2 to 4 cm^2^ of surface, over about 6–10 s. Samples (n = 6 for each condition, situated at decimetric to metric distance from one another in any given location) were placed immediately into liquid nitrogen and later transferred at -80 °C until DNA extraction. For electron microscopy, samples were taken in March 2021 with a small chisel, and authorization for sampling was granted only for masonry benches. Bulls center and Passage vertical were studied, and samples were placed in tubes containing 0.1 M sodium cacodylate buffer with 2% glutaraldehyde and kept à 4 °C.

### DNA extraction and high-throughput sequencing

Extraction of total DNA was carried out with the FastDNA SPIN kit for Soil (MP Biomedicals, Illkirch, France), following the manufacturer’s instructions. The lysis solution was added to the tube containing the sample matrix. Elution was performed using 80 µL volume for each sample, and the final DNA concentration was measured using the Qubit dsDNA Hs Assay Kit (Invitrogen, Carlsbad, USA) following the manufacturer’s instructions. The DNA extracts were kept at − 20 °C.

Three gene markers were analyzed in each sample. The V3-V4 region of the 16S rRNA genes of Bacteria was amplified using the universal primers 341F/805R [24], whereas the V3-V4 region of the 16S rRNA genes of Archaea was amplified using the universal primers 515F/915R [25]. The fungal ITS2 region was amplified using the universal primers ITS3_KYO2/ITS4 [26] (Additional file 1: Table S1). The PCR mix consisted in 5 µL of 5 × Hot BioAmp Blend Master Mix RTL (Biofidal, Vaulx-en-Velin, France), 0.1 µM of each primer, 0.1 × of GC-rich-enhancer (Biofidal), 0.2 ng.µL^−1^ of Bovine Serum Albumin (Promega, Madison, USA) and 0.2–1.0 ng of template DNA. All amplifications were performed in triplicate, in a Bio-Rad T1000 thermal cycler (Bio-Rad, Hercules, USA). The PCR program for Bacteria was 3 min at 95 °C, 28 cycles of 45 s at 95 °C, 45 s at 50 °C and 90 s at 72 °C, followed by 7 min at 72 °C. For Archaea, it consisted in 10 min at 94 °C, 30 cycles of 1 min at 94 °C, 1 min at 58 °C and 90 s at 72 °C, followed by 10 min at 72 °C. For Fungi, PCR was done with 10 min at 95 °C, 28 cycles of 20 s at 94 °C, 30 s at 47 °C and 20 s at 72 °C, followed by 7 min at 72 °C. Primers were tagged with the Illumina adapters TCG TCG GCA GCG TCA GAT GTG TAT AAG AGA CAG and GTC TCG TGG GCT CGG AGA TGT GTA TAA GAG ACA G, enabling a two-step PCR construction of amplicon libraries. DNA extraction was also carried out without any biological matrix and this was considered a negative control to evaluate ambient contamination. Amplicons were checked by electrophoresis on 1.5% agarose 20 min at 100 V and UV visualization, and the correct lengths were obtained with samples (and no signal with the blanks, as expected). Illumina MiSeq (2 × 300 bp, paired-end chemistry v3) was done after pooling PCR triplicates, and was performed by Biofidal (Lyon, France), aiming (for each sample) at 40,000 sequences for the bacterial 16S rRNA gene and the ITS, and 70,000 sequences for the archaeal 16S rRNA gene (as certain bacterial 16S sequences—later discarded—amplify as well).

### Bioinformatic treatment of Illumina sequence data

For each of the three datasets (i.e. bacterial 16S rRNA genes, archaeal 16S rRNA genes and fungal ITS2), paired-end reads were demultiplexed in the different samples according to exact match adaptors (subsequently removed). The reads obtained were merged using Fast Length Adjustment of Short reads (FLASh) [27], based on a maximum of 10% mismatches in the overlap region. Denoising was carried out by discarding reads without the expected 200–500 bp length or that displayed ambiguous bases (N). Once sequence dereplication was done, clusterization was performed using SWARM [28], based on a local clustering threshold (rather than a global threshold) and an aggregation distance of 3 for identification of operational taxonomic units (OTUs). Chimeric OTUs were discarded using VSEARCH [29] and sequences of low abundance were filtered at 0.005% of all sequences [30]. OTU affiliation was carried out with both RDP Classifier and BLASTn [31] against the 138.1 SILVA database [32] for bacterial and archaeal 16S rRNA genes and the 8.2 UNITE database for fungal ITS markers [33], which was automated in the FROGS pipeline [34]. Contaminant OTUs identified from the negative controls (blanks) samples were removed. Normalization for sample comparison was implemented by randomly resampling down to 13,467 and 12,753 sequences in the bacterial and fungal datasets, respectively, whereas the archaeal dataset was not normalized due to very low numbers of reads in dark zones.

### Statistical analysis

The efficacy of sampling and sequencing was evaluated using rarefactions curves. Alpha diversity at OTU level was measured with Chao-1 index [35], Shannon’s H’ index [36], Evenness index [36] and Simpson 1-D index [37], using Paleontological Statistics (PAST) software (version 4.04) [38]. The diversity indices were assessed with Kruskal–Wallis tests and post-hoc Wilcoxon pairwise tests, or with ANOVA and post-hoc Tukey-HSD using ‘vegan’ package in R (version 4.0.3) [39]. Comparison of microbial community composition was performed using non-metric multidimensional scaling (NMDS) based on abundance dissimilarity matrices (Bray–Curtis) and sample ordination. This analysis was carried out using the R package 'phyloseq' [40–42]. Replicates of each situation (i.e. room and rock surface condition) were grouped into one condition, and NMDS was vectorized from unmarked surface (control) to altered surface (dark zone), which was done using ‘phyloseq’, ‘vegan’ and ‘dplyr’ packages in R [39, 40, 43]. The stress value was calculated to measure the difference between the ranks on the ordination configuration and the ranks in the original similarity matrix for each replicate. Stress values below 0.1 are considered without risk and those not exceeding 0.2 are acceptable [44]. Then, analysis of variance using distance matrices (Adonis) was performed using ‘vegan’ and ‘pairwiseAdonis’ packages, to assess differences at *P* < 0.05 in overall microbial community composition. A Holm-Bonferroni correction was applied on *P* values to lower alpha risk. In addition, correlations between the date of dark zone appearance and Bray–Curtis community distance were assessed using Kendall's correlation coefficient τ. All statistical analyses were performed using R software (version 4.0.3) [45].

### Quantitative PCR

To assess the number of bacterial 16S rRNA genes, archaeal 16S rRNA genes and fungal ITS2 regions, quantitative PCR (qPCR) was performed using primers 519F (5′-CAGCMGCCGCGGTAANWC-3′)/907R (5′-CCGTCAATTCMTTTRAGTT-3′; 400-bp amplicon) [46], 787F (5′- ATTAGATACCCSBGTAGTCC-3′)/1059R (5′-GCCATGCACCWCCTCT-3′; 210-bp amplicon) [47] and ITS7F (5′-GTGYCAGCMGCCGCGGGTA-3′)/ITS4R (5′-TCCTCCGCTTATTGATATGC-3′; 149-bp amplicon) [48], respectively. Briefly, qPCR assays were conducted in triplicate using 10 µL of LightCycler 480 SYBR Green I Master mix, 2 µL of sample DNA, 2 µL (final concentration 0.3 µM) of each primer in a final volume of 20 µL, with a thermocycling CFX-96 Connect (BioRad, USA). The standard curves were generated using plasmid DNA containing one copy of 16S rRNA or 18S rRNA gene, after dilution from 10^8^ to 10^3^ copies.µL^−1^. Each standard curve point was done in three replicates for each qPCR plate. PCR was done with denaturation at 95 °C for 10 min, followed by 40 cycles of 15 s (bacteria) or 30 s (fungi) denaturation at 95 °C, hybridization 60 s at 63 °C (Bacteria) or 45 s at 57 °C (Fungi), and 30 s (Bacteria) or 90 s (Fungi) elongation at 72 °C. For Archaea, PCR was done with denaturation at 37 °C for 10 min and 95 °C for 15 min, followed by 45 cycles of 15 s denaturation at 95 °C and 60 s hybridization at 60 °C. The melting curve was made by measuring the fluorescence during a temperature increase from 72^◦^C to 95 °C, in increments of 0.5 °C every 10 s. Melting curve calculation and Tm determination were done using the Tm Calling Analysis module of CFX Maestro Software v 2.3 (BioRad). Results were obtained as numbers of copies. They were normalized to the total DNA quantity extracted and expressed as numbers of copies per ng of total DNA. To verify possible bias linked to the presence of qPCR inhibitors, two samples for each targeted gene were diluted at 1/10, 1/50, 1/100 and 1/200. A curve was drawn for each sample to determine the dilution range in which the efficiency of amplification was between 80 and 100% (cycle threshold according to amplicon concentration).

### Definition of dark zone reference criteria based on Apse data

For the three domains of life, a range of criteria were defined for subsequent quantification of similarity level with the Apse’s dark zones. First, genus-centered criteria were computed for Bacteria and Fungi, as follows. For the Apse’s dark zone and control, the mean numbers of reads for individual genera were log transformed, and the variation in relative abundance between limestone (control) and dark zone was calculated as Δlog = log(control) − log(dark zone). All taxa with a Δlog value > 1 or < − 1 (arbitrarily-chosen thresholds) were selected as reference criteria, making 41 reference taxa. Second, the number of reads for Archaea were log-transformed, and the variation between control and dark zone was calculated as Δlog = log(control) − log(dark zone). Third, the Bray–Curtis distance between control and dark zone was calculated separately for Bacteria and Fungi. Fourth, qPCR levels for Bacteria, Archaea and Fungi (expressed as numbers of gene copies per ng of extracted DNA) were also used.

### Construction of the decision matrix

**Fig. 2:**
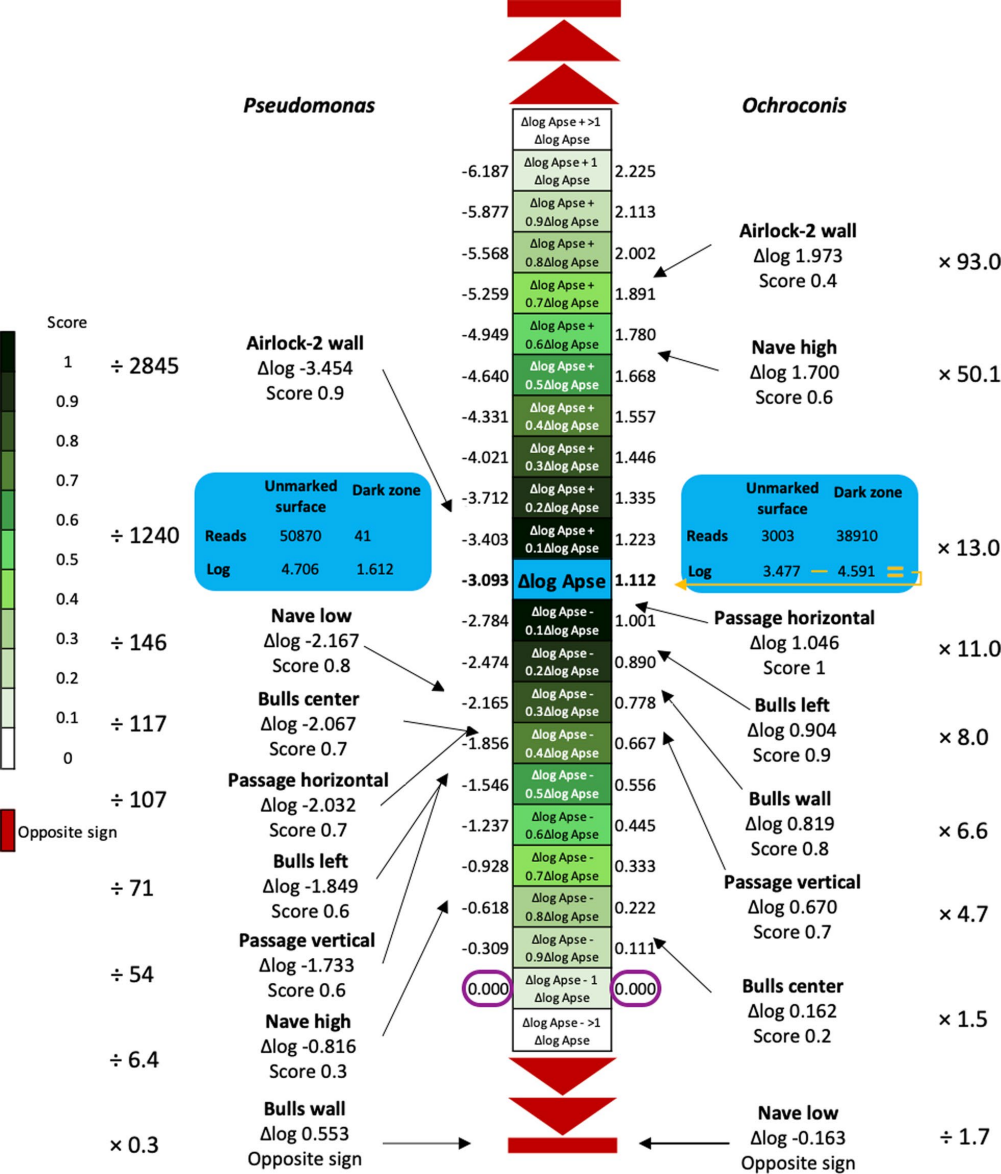
Determination of scores assigned for each similarity criterion based on their Δlog values i.e. [Δlog = log(control) − log(dark zone)] with the data obtained for the Apse reference situation (in blue). The calculation is shown in orange for *Ochroconis* in the Apse. The data (higher or lower) obtained for the other dark zones are positioned according to the different intervals of values, each corresponding to a given scale of gap with the Apse reference situation, resulting into a score between 0 and 1 (with increments of 0.1, according to the green gradient), whereas data for which the gap falls outside of the scale or with an opposite sign are represented in red (meaning that the difference in the number of sequences between the two rock surface conditions is greater than those indicated in the scale). The Δlog value are computed, and the fold change value between this Δlog value and the corresponding Δlog value for the Apse (i.e., ΔFold-change) is scored as 1 (when |Δfold change| is below one tenth of the Δlog value for the Apse), 0.9 (when |Δfold change| is between one and two tenths of the Δlog value for the Apse), …, and 0 (when |Δfold change| is above nine tenths of the Δlog value for the Apse). When the Δlog value is equal to 0.000 (framed in mauve), it means that the number of sequences is the same for both rock surface conditions (i.e. control and dark zone). Δlog values and score assignations are illustrated for *Ochroconis* and *Pseudomonas* criteria in all sample locations. The division factor (when counter-selection) or multiplication factor (when selection) for the number of sequences between the two rock surface conditions is indicated on the side, to facilitate reading

To determine the extent to which dark zones from different Lascaux rooms correspond to those present in the Apse, a decision matrix was built on the basis of the reference criteria defined above, using data from each dark zone studied (and its corresponding control). Computation was done as follows (illustrated in Fig. 2 for *Ochroconis* and *Pseudomonas* genera). First, for each of the 41 reference taxa, the Δlog value was computed (Additional file 1: Table S2), and the fold change value between this Δlog value and the corresponding Δlog value for the Apse (i.e., ΔFold-change) was scored as 1 (when |Δfold change| was below one tenth of the Δlog value for the Apse), 0.9 (when |Δfold change| was between one and two tenths of the Δlog value for the Apse), …, 0.1 (when |Δfold change| was between eight and nine tenths of the Δlog value for the Apse) and 0 (when |Δfold change| was above nine tenths of the Δlog value for the Apse) (Fig. 2). The comparisons were implemented using a script in Python version 3.10.1 [49]. Second, the same approach was followed with the number of archaeal reads. Third, for the criterion based on the Bray–Curtis distance (computed with ‘vegdist’ package in R [39]), a measurement of the difference between the Apse and each of the other situations studied was done, by computing (i) ⍺ = Bray–Curtis distance between the controls in the Apse and the controls in the other sampling areas, (ii) β = Bray–Curtis distance between the dark zones in the Apse and the dark zones in the other sampling areas, and (iii) (β-⍺)/⍺ representing the differential dark zone evolution between the Apse and the other sampling areas. On this basis, a decimal score was obtained, ranging from 1 (when (β-⍺)/⍺ < 0.1) to 0 (when (β-⍺)/⍺ > 0.9). Finally, the sum of the 44 scores was computed.

### Scanning electron microscopy

Glutaraldehyde-fixed samples were serially washed in 0.1 M sodium cacodylate baths over 2 days, and were dehydrated using ethanol solutions at 30%, 50%, 70%, 80%, 95% (30 min each), and 100% ethanol (three times 30 min). The samples were placed in a mixture of 50% ethanol and 50% hexamethyldisilazane for 30 min and again for 60 min (twice), and in 100% hexamethyldisilazane overnight, till total evaporation was achieved. The samples were then metalized with carbon and analyzed by Scanning Electron Microscopy (SEM) using a FEG FEI Quanta 250 SEM microscope equipped with an Everhart–Thornley Detector (ETD, secondary electrons) (FEI Company, Hillsboro, USA).

## Results

### Microbial community structure on unmarked rock surfaces

**Fig. 3:**
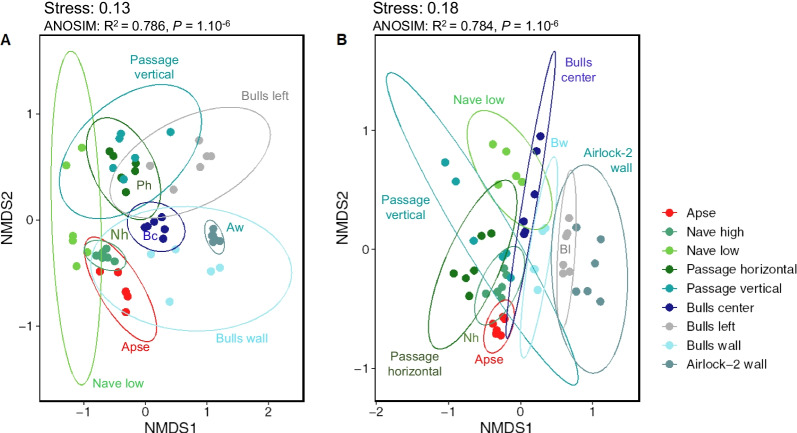
Non-metric multidimensional scaling (NMDS) analysis of microbial community structure in unmarked surfaces of Lascaux Cave according to sampling position (i.e. location in the cave). Ellipses (95% confidence intervals) indicate the different sampling positions for bacterial (**A**) and fungal (**B**) communities. Aw, Airlock-2 wall; Bc, Bulls center; Bl, Bulls left; Nh, Nave high; Ph, Passage horizontal

NMDS and ANOSIM tests indicated that the effect of the sampling location on community structure was significant on unmarked surfaces for Bacteria (*P* = 0.001, R^2^ = 0.70, Fig. 3A) and Fungi (*P* = 0.007, R^2^ = 0.63, Fig. 3B), meaning that Lascaux’s microbiota is spatially heterogeneous (Additional file 1: Table S3).

For Bacteria on unmarked surfaces, Bulls left (R^2^ = 0.60), Bulls wall (R^2^ = 0.51) and Nave low (R^2^ = 0.47) showed significant differences compared with the Apse (*P* = 0.036) (Additional file 1: Table S3). Bacterial communities were different between Passage horizontal and Passage vertical (*P* = 0.036, R^2^ = 0.28), but significance level was only *P* = 0.072 for Bulls wall compared with Airlock-2 wall (R^2^ = 0.41), Nave low (R^2^ = 0.44) and Passage horizontal (R^2^ = 0.52).

For Fungi on unmarked surfaces, the Apse differed from Bulls wall (*P* = 0.036, R^2^ = 0.53) but significance level was only *P* = 0.072 for the Apse vs Airlock-2 wall (R^2^ = 0.46). In addition, there was also a trend (*P* = 0.072) for a difference between Bulls center and Nave high (R^2^ = 0.54), Passage horizontal (R^2^ = 0.38), Passage vertical (R^2^ = 0.37) and Airlock-2 wall (R^2^ = 0.71) (Additional file 1: Table S3).

### Microbial community composition on unmarked rock surfaces

**Fig. 4:**
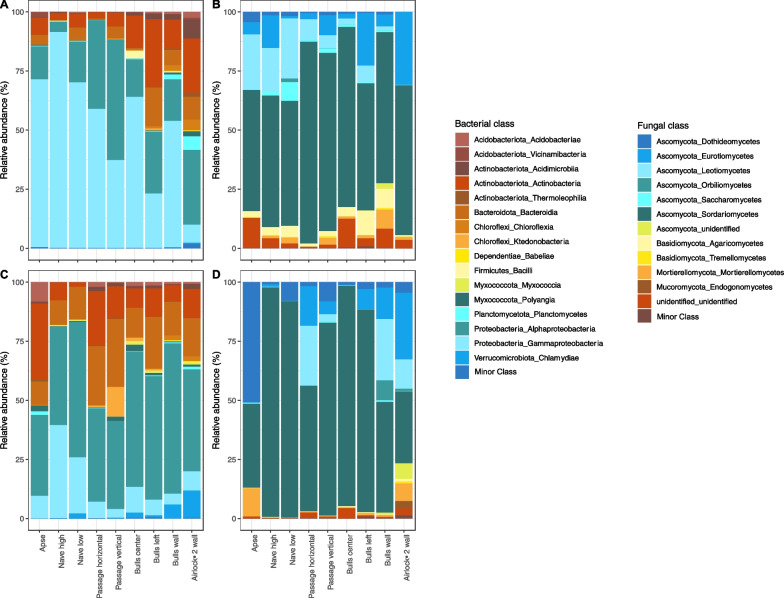
Relative abundance (% of sequences) of bacterial (**A**, **C**) and fungal (**B**, **D**) classes for the controls (**A**, **B**) and dark zones (**C**, **D**). Phyla or classes of relative abundance < 0.1% were considered as minor phyla or minor classes, respectively

The bacterial community was composed of 16 to 24 classes (depending on location) on unmarked surfaces, with high variations for the most abundant classes Gammaproteobacteria (from 7.7 to 91.3% of sequences), Alphaproteobacteria (4.4%-51.0%) and Actinobacteria (2.8–22.9%) (Fig. 4A). Notably, the Apse differed from Passage vertical, Bulls left and Airlock-2 wall, as follows. The Actinobacteria amounted to 7.2% in the Apse versus 28.6% in Bulls left and 23.0% in Airlock-2 wall, and the Alphaproteobacteria to 14.0% versus 51.0% in Passage vertical, 26.0% in Bulls left and 31.6% in Airlock-2 wall. The Planctomycetes reached 5.75% and Acidimicrobiia 8.25% in Airlock-2 but lower values (< 0.1–0.3% and 0.1–2.2%, respectively) in the other locations, whereas the Gammaproteobacteria reached only 7.6% in Airlock-2 versus 37.1–91.3% elsewhere. The Bacteroidia accounted for 16.4% of sequences in Bulls left versus only < 0.1–6.5% in the other locations, and the Bacili 3.1% in Bulls center versus < 0.1–0.4% elsewhere.

The fungal community was composed of 12–17 classes on unmarked surfaces. The Sordariomycetes (51.3–76.2%), Eurotiomycetes (1.02–30.0%) and Leotiomycetes (0.09–25.0%) were the most abundant classes on all unmarked surfaces (Fig. 4B). Compared with other locations, the reference area (Apse) and Nave displayed a lower proportion of Sordariomycetes (respectively 51.3% and 55.2%, versus 63.4–85.4% elsewhere) and higher proportions of Leotiomycetes (respectively 23.3% and 18.4%, versus < 0.1–9.0%) and Dothideomycetes (respectively 4.4% and 3.0%, versus 0.1–1.8%). The Agaromycetes reached 8.1% and Mortierellomycetes 8.0% in Bulls wall but lower values (0.4–3.3% and < 0.1–2.5%, respectively) in the other locations. Nave low and Bulls left showed a higher proportion of Eurotiomycetes (13.8% and 22.3%, respectively) compared with the other locations (0.9–8.4%). The Leotiomycetes accounted for 1.6% of sequences in Bulls wall and 0.09% in Airlock-2 versus 6.1–25.4% in the other locations, and the Saccharomycetes 8.0% in Nave low but only 0.1–1.6% elsewhere (Fig. 4B).

### Microbial community size on unmarked surfaces

The effect of sampling location on community size (qPCR) on unmarked surfaces differed for Bacteria, Archaea and Fungi (Additional file 1: Table S4 and Fig. S1). For Bacteria, community size in the Apse differed significantly from all other sample locations excepted Airlock-2 wall (*P* = 0.42). In addition, it was also different between Nave low and Nave right (*P* = 0.001), and between Passage vertical and Passage horizontal (*P* = 0.001). For Archaea, community size was higher in the Apse than in all other sample locations (all *P* = 0.001). It also differed between each sample locations (all at *P* ≤ 0.001) excepted between Nave high and Nave low (*P* = 0.47). For Fungi, community size was lower in the Apse compared with all other sample locations (all at *P* ≤ 0.005), and all sample locations differed from one another (all at *P* ≤ 0.001).

### Microbial community structure of dark zones compared with unmarked surfaces

**Fig. 5:**
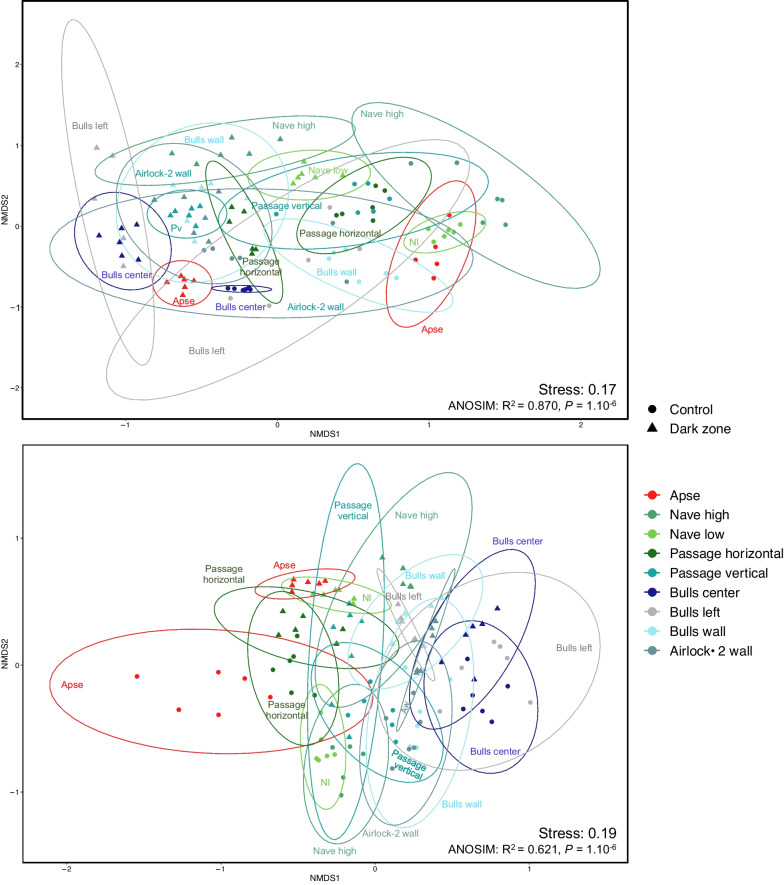
Non-metric multidimensional scaling (NMDS) analysis of microbial community structure in Lascaux Cave according both to sampling position (i.e. location in the cave) and rock surface condition (i.e. control or dark zone). Controls and dark zone are represented by circles and triangles, respectively. Ellipses (95% confidence intervals) indicate the different sampling positions for bacterial (**A**) and fungal (**B**) communities. Aw, Airlock-2 wall; Nl, Nave low; Pv, Passage vertical

NMDS and ANOSIM tests indicated that the effect of rock surface condition (control vs dark zone) on community structure was significant for Bacteria (*P* = 0.009, R^2^ = 0.17, Fig. 5A) and Fungi (*P* = 0.009, R^2^ = 0.08, Fig. 5B). The interaction between location in Lascaux Cave and rock surface condition was significant for bacterial (*P* = 0.001, R^2^ = 0.23) and fungal communities (*P* = 0.001, R^2^ = 0.18) (Additional file 1: Table S5).

For both taxonomic markers, dark zones differed from their adjacent unmarked surfaces at each location in Lascaux Cave (Additional file 1: Table S5), and the interaction between location and rock surface condition was significant both for bacterial (*P* = 0.001, R^2^ = 0.23) and fungal communities (*P* = 0.001, R^2^ = 0.18). For the bacterial community, the difference between dark zone and control was the largest in the Apse (*P* = 0.001, R^2^ = 0.80) and the smallest in Bull left (*P* = 0.011, R^2^ = 0.24). For the fungal community, the difference between dark zone and control was of less significance in Passage horizontal (*P* = 0.011, R^2^ = 0.29), Passage vertical (*P* = 0.005, R^2^ = 0.27) and Airlock-2 (*P* = 0.009, R^2^ = 0.22) than in other locations (all at *P* = 0.003, R^2^ = 0.39–0.72).

### Microbial community diversity of dark zones compared with unmarked surfaces

Diversity indices (Simpson 1-D, Shannon H’, Evenness index and Chao-1) were calculated for bacterial and fungal communities (Additional file 1: Table S6). First, the Simpson’s index for bacteria was statistically lower in control than dark zone samples (Wilcoxon test: *P* = 0.026), but differences were not significant for fungi (*P* = 0.347). Second, the Shannon H’ index was similar for all rock surface conditions, for the bacterial (*P* = 0.418) and the fungal communities (*P* = 0.326). Third, the Evenness index for bacteria was lower in control than dark zone (*P* = 0.023), but differences were not significant for fungi (*P* = 0.458). Fourth, the Chao-1 index was similar for all rock surface conditions, for the bacterial (*P* = 0.782) and the fungal communities (*P* = 0.068).

### Microbial community composition of dark zones compared with unmarked surfaces

The Bray–Curtis distances for the bacterial and fungal communities were computed between control and dark zone samples for each room. Kendall's correlation between the date of dark zone appearance (relative to the starting date in the Apse) and the Bray–Curtis distance was negative and not significant for the bacterial (τ = − 0.34, *P* = 0.21) and the fungal communities (τ = − 0.55,* P* = 0.051) (Additional file 1: Fig. S1).

Bacterial classes of Gammaproteobacteria, Alphaproteobacteria, Bacteroidia and Actinobacteria showed the most important variations (positive or negative) between unmarked and dark zone conditions overall (− 40%, + 23%, + 12% and + 1.8%, respectively) (Fig. 4A–C). When considering locations, the highest variation between unmarked surface and dark zone was observed for Gammaproteobacteria in the Apse (71.0% in dark zone vs 9.4% in the control), whereas this class reached similar levels in Airlock-2 wall (8.1% vs 7.6%, respectively) (Fig. 4A–C). The Alphaproteobacteria were at higher level in dark zone in Bulls wall (57.2% in dark zone vs 15.8% in the control), unlike in Passage vertical (37.1% in dark zone vs 50.9% in the control). The Bacteroidia displayed higher levels in dark zone compared with unmarked surface, notably in Passage horizontal (24.7% in dark zone vs 0.6% in the control) and Passage vertical (28.2% vs 5.1%, respectively), as found with the Chlamydiae in Bulls walls (2.1% in dark zone vs 0.1% in the control) and Airlock-2 wall (11.4% vs 0.5%, respectively). The Actinobacteria reached higher levels in dark zones in the Apse (32.1% in dark zone vs 7.2% in the control), Passage horizontal (23.1% vs 2.7%, respectively) and Passage vertical (13.5% vs 5.6%, respectively), but the opposite was found in Bulls left (8.1% in dark zone vs 13.6% in the control), Airlock-2 wall (12.3% vs 22.9%, respectively), Bulls wall (6.8% vs 12.4%, respectively) and Bulls center (12.1% vs 28.6%, respectively).

Variations (positive or negative) between dark zone and unmarked surface were of lower magnitude for fungal classes, the most important ones concerning the Dothideomycetes (+ 7.6%), Sordariomycetes (+ 4.2%), Agaromycetes (− 3.8%) and Leotiomycetes (− 2.9%) (Fig. 4B–D). Taking into consideration the various sampling locations within Lascaux, the Dothideomycetes were at higher level in dark zone in the Apse (50.9% in dark zone vs 4.4% in the control), unlike in Nave high (0.9% and 1.5%, respectively) (Fig. 4B–D). The Leotiomycetes were in higher amounts in dark zones for Passage horizontal (25.2% in dark zone vs 8.9% in the control), Bulls wall (26.1% vs 1.6%, respectively) and Airlock-2 wall (12.4% vs 0.1%, respectively), but the opposite was found for Apse (0.1% in dark zone vs 23.3% in the control), Nave high (0.2% vs 18.4%, respectively), Nave low (< 0.1% vs 25.4%, respectively) and Bulls center (< 0.1% vs 7.1%, respectively).

### Microbial community size of dark zones compared with unmarked surfaces

The size (qPCR) of the bacterial community was significantly higher in dark zones compared with unmarked surfaces for all locations excepted Airlock-2 wall (*P* = 0.49) and Bulls left (*P* = 0.47) (Additional file 1: Table S7 and Fig. S2), while archaeal community size was higher in unmarked surfaces compared with dark zones for all sample locations (all at *P* = 0.001). Fungal community size was higher in dark zones compared with unmarked surfaces for all locations (all at* P* ≤ 0.001) excepted in Bulls wall, where it was higher in unmarked surface compared with dark zone (*P* = 0.003) (Additional file 1: Table S8 and Fig. S2).

**Fig. 6:**
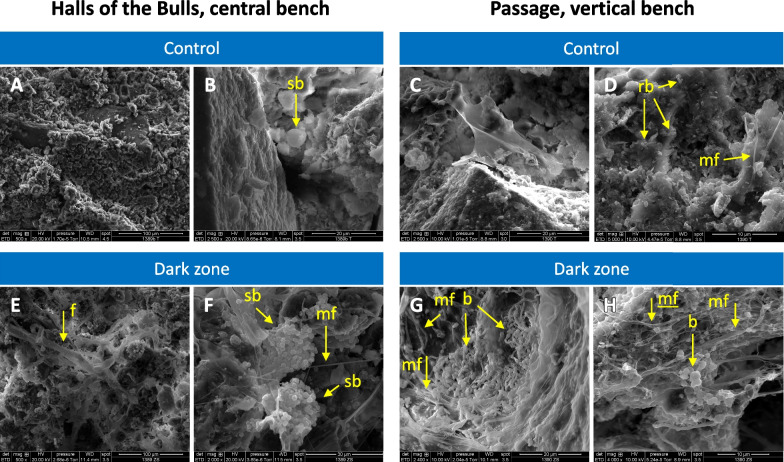
Scanning electron microscopy pictures of dark zones (**E**–**H**) and unmarked surfaces nearby (**A**–**D**) at various magnifications, in the Hall of the Bulls (central bench) and the Passage (vertical bench). Spherical bacteria are indicated by *sb* (one in **B**, many in **F**), bacteria of various shapes by *b* (in **G** and **H**), fungi by *f* (in **E**), and microbial filaments by *mf* (in **D**, **F**–**H**)

The occurrence of microorganisms was further assessed based on scanning electron microscopy, at the only locations where we were allowed to take samples using a blade (small chisel). Bacteria or Archaea could be seen outside of dark zones, essentially as individual cells, and a few filaments were also found (illustrated in Fig. 6D). In contrast, prokaryote cells were readily observed in dark zone samples, sometimes as individual cells or small clusters of a few cells, but more often as large clusters of many more cells (illustrated in Fig. 6E–H). Filamentous microorganisms were seen (e.g. fungal filaments in Fig. 6E). Filaments likely produced by microorganisms (putatively polysaccharides or fimbriae; Fig. 6D,F–H) were sometimes observed in the vicinity of microorganisms. Overall, qualitative observations of samples from Passage vertical and Bulls central supported the higher levels of prokaryotes and Fungi in dark zones that were determined by qPCR.

### Comparisons with reference dark zone from the Apse

**Fig. 7:**
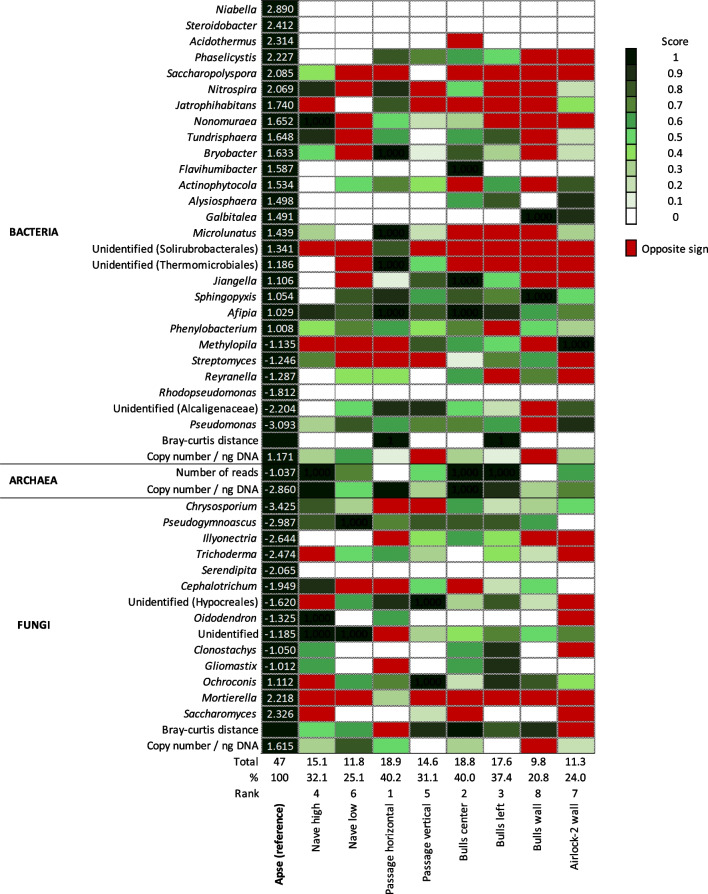
Decisional matrix based on the dark zone reference (in the Apse) for 47 criteria corresponding to 27 bacterial genera, 14 fungal genera, 1 bacterial and 1 fungal Bray–Curtis distance, the number of archaeal reads, and the copy number of marker genes (per ng of DNA) for Bacteria, Archaea and Fungi. The Δlog Apse value is indicated in white (when applicable). Scores between 0 and 1 are shown with a green color intensity (gradient for each 0.1 increment; see “Materials and Methods section” for more details), and red is used when changes were opposite to those for the Apse (i.e. Δlog was of opposite sign compared with Δlog Apse). The total score as well as the % of similarity to the dark zone reference were calculated for each dark zone, and the ranking based on the % similarity is indicated. Detailed calculation is provided in Additional file 1: Table S9

When the decisional matrix was built to assess similarity level of various dark zones to the reference dark zone in the Apse, 5 of the 47 criteria (i.e. for the bacterial genera *Niabella*, *Steroidobacter*, *Acidothermus*, *Rhodopseudomonas* and fungal genus *Serendipita*) were scored 0 in all other dark zones whereas similarities were found for the 42 other criteria (Fig. 7; Additional file 1: Table S9). When considering the three domains of life together, Passage horizontal, Bulls center, Bulls left, Nave high and Passage vertical obtained global scores of respectively 18.9, 18.8, 17.6, 15.1 and 14.6 when adding positive scores for the 47 criteria, i.e. similarities of respectively 40.2%, 40.0%, 37.4%, 32.1% and 31.1% to the reference dark zone in the Apse based on the 47 criteria (Fig. 7). Scores for Nave low, Airlock-2 wall and Bulls wall were only 11.8, 11.3 and 9.8, respectively, i.e. similarities of respectively 25.1%, 24.0% and 20.8%.

Some of the criteria are of particular interest, e.g. because they displayed large variations or were identified as potentially important to explain dark zone formation [22]. For the Bray–Curtis distance criterion, contrasting results between bacterial and fungal communities were observed (Fig. 7; Additional file 1: Table S9). For the bacterial community, two locations (Passage horizontal and Bulls left) displayed similar community changes related to the formation of dark zones when compared with the Apse (i.e. with a score of 1), while the other locations had a score of 0. For the fungal community, the highest score was observed for Nave low (with a score of 0.8) and the lowest scores for Passage vertical and Bulls left (with a score of 0) and especially for Bulls wall (with an opposite variation).

For the bacterial community (Fig. 7; Additional file 1: Table S9), the *Pseudomonas* genus was the bacterial criterion with the biggest variation in the Apse (Δlog Apse = − 3.093); except for Bulls wall (opposite sign), all other dark zones showed also *Pseudomonas* counter-selection, with a score of 0.3 in Nave high to 0.9 in Airlock-2 wall (an average of 0.66). *Sphingopyxis* and *Afipia* genera displayed moderate variation in the Apse (Δlog Apse = 1.054 and 1.029, respectively), and the eight other dark zones had the same variation sign with high score 0.5 to 1 (excepted for Nave high with score of 0) for *Sphingopyxis* and 0.6 to 1 for *Afipia*. For genera *Nonomuraea* (Δlog Apse = 1.652) and *Bryobacter* (Δlog Apse = 1.633), thought to play a role in dark zone evolution [22], we found (i) high scores in Nave high (1 and 0.5, respectively) and Passage horizontal (0.5 and 1, respectively), (ii) moderate scores in Bulls back (0.3 and 0.8, respectively) and Passage vertical (0.2 and 0.1, respectively), and (iii) on the contrary an opposite variation (i.e. with opposite sign) in Nave low and Bulls wall. At the entire bacterial community level, the Bray–Curtis distance gave scores of 1 (Passage horizontal and Bulls left), 0.9 (Passage vertical), 0.8 (Bulls wall), 0.7 (Bulls back), 0.5 (Nave low) and 0 (Nave high and Airlock-2 wall). qPCR data for bacteria gave a similarity score of 0.6 for Nave low, 0.3 for Nave high, Bulls center and Airlock-2 wall, 0.1 for Passage horizontal and Bulls left, but 0 for Passage vertical and the variation was even opposite for Bulls wall.

For the archaeal community, only the Δlog number of reads (with Δlog Apse = − 1.037) and the copy number of archaeal 16S rRNA genes (Δlog Apse = − 2.860) were considered to assign similarity scores, because of the strong counter-selection of Archaea in dark zones (Fig. 7; Additional file 1: Table S9). Nave high and Bulls center had a same variation (with scores > 0.9) for both criteria, Nave low and Airlock-2 wall showed scores between 0.5 and 0.7, whereas Passage horizontal and Bulls wall had a score of 0 for the number of reads and 1 and 0.3 (respectively) for qPCR levels.

For the fungal community (Fig. 7; Additional file 1: Table S9), the *Pseudogymnoascus* genus (Δlog Apse = − 2.987) had a score of 1 in Nave low, 0.8 in Nave high, Passage vertical, Bulls back and Bulls left, 0.7 in Passage horizontal and 0.6 in Bulls wall, but of 0 in Airlock-2 wall. The black-melanized fungal genus *Ochroconis* proliferating in the Apse’s dark zones (Δlog Apse = 1.112) was also observed in all other dark zones excepted in Nave high; Passage vertical, Bulls left, Bulls wall and Passage horizontal had scores of respectively 1, 0.9, 0.8 and 0.7, and Airlock-2 wall and Bulls center only of 0.4 and 0.2, respectively. *Mortierella* (Δlog Apse = 2.218) gave a score of 0.3 in Passage horizontal but all the other dark zones displayed an opposite variation. The *Chrysosporium* genus was the fungal criterion with the biggest variation (Δlog Apse = − 3.425), and the other dark zones had scores between 0 and 0.8 or displayed an opposite variation (for Passage). For the Bray–Curtis distance criterion, Bulls center, Passage vertical, Bulls wall, Bulls left, Nave high and Nave low had scores of 1, 0.9, 0.9, 0.8, 0.6 and 0.5, respectively, whereas Passage horizontal and Airlock-2 wall had scores of 0. With qPCR data of fungi, scores were 0.7 for Nave low, 0.5 for Passage vertical, 0.3 for Nave high and Bulls center, 0.2 for Airlock-2 wall, 0 for Passage vertical and Bulls left, but the variation was opposite for Bulls wall.

### Cosmopolitan taxa vs taxa specific and/or endemic to dark zones

**Fig. 8:**
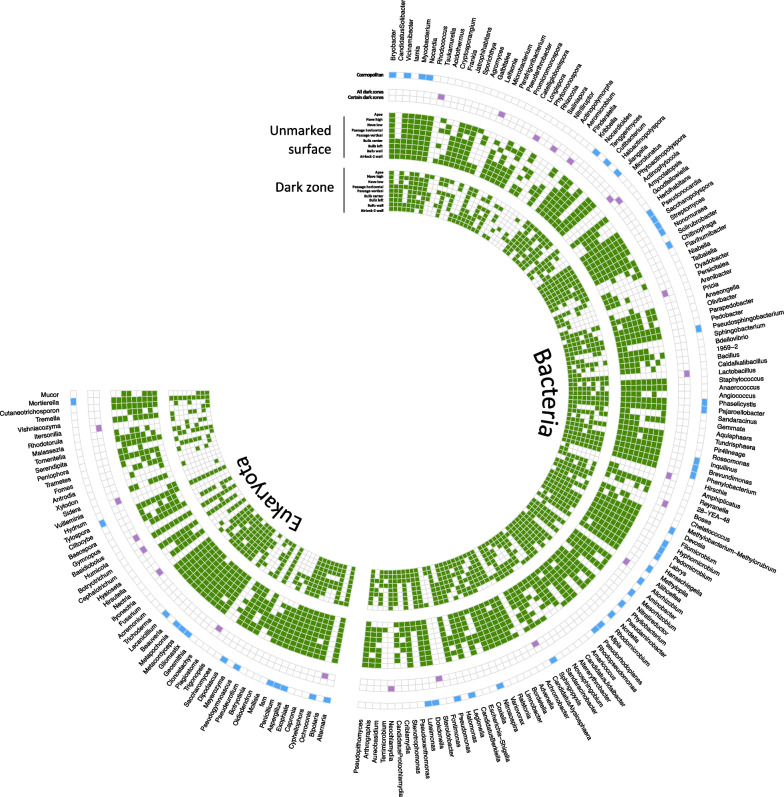
Occurrence of bacterial and fungal genera in unmarked surface and dark zone across nine sample locations in the Lascaux Cave. Presence of these genera in each condition is represented by green squares. In the outer circles, blue indicates cosmopolitan genera i.e. present in all dark zone samples and all unmarked surface samples in all locations (‘Cosmopolitan’), and purple the dark zone-specific genera found in all dark zone samples in all locations (‘All dark zones’) and dark zone-specific genera found in all samples of certain dark zone locations (‘Certain dark zones’)

Out of the 197 genera evidenced here, 50 cosmopolitan genera (36 Bacteria and 14 Fungi) were identified for unmarked surfaces and dark zones together (Fig. 8). Taxa were considered as cosmopolitan when present in all replicates of both types of samples at each of the nine locations in the cave. They included taxa already documented in Lascaux Cave [21, 22], such as the bacterial genera *Pseudomonas* (amounting here to an average of 20.7% of all bacterial sequences across the nine locations)*, Chitinophaga* (6.6%), *Afipia* (2.9%)*, Nonomuraea* (1.5%), *Bryobacter* (1.0%) and *Labrys* (0.9%), and the fungal genera *Ochroconis* (representing here an average of 4.9% of fungal sequences), *Pseudogymnoascus* (5.1%)*, Mortierella* (2.2%) and *Gliomastix* (0.4%).

Six dark zone-specific taxa (i.e. taxa present in dark zones at all nine locations and not detected in any unmarked surface sample) were identified (Fig. 8). All of them were bacteria and occurred at low relative abundances, i.e. *Actinophytocola* (amounting to an average of 0.7% of all bacterial sequences from Lascaux dark zones)*, Neochlamydia* (0.6%), *Bosea* (0.5%)*, Microbacterium* (0.1%)*, Tsukamurella* (0.1%) and *Lactobacillus* (< 0.1%). In addition, a few dark zone-specific taxa endemic to a particular sampling location were found for 5 of the 9 sampling locations, again at low relative abundances, i.e. the Apse (the fungi *Bipolaris* [amounting to < 0.1% of fungal sequences at this location]*, Xylodon* [< 0.1%] and *Itersonilia* [< 0.1%]), Nave high (the bacteria *Phytomonospora* [amounting to 0.9% of bacterial sequences at this location], *Achromobacter* [< 0.1%], and the fungi *Plagiostoma* [0.1%] and *Nectria* [< 0.1%]), Bulls center (the bacteria *Nitriliruptor* [< 0.1%] and *Phytoactinopolyspora* [< 0.1%]), Bulls left (the bacteria *Arenibacter* [0.2%], *Flindersiella* [0.1%] and *Aliihoeflea* [< 0.1%], and the fungus *Basidiobolus* [< 0.1%]) and Bulls wall (the bacteria *Hirschia* [< 0.1%] and the fungus *Vulleminia* [0.1%]). No dark zone-specific, endemic taxa were observed in Nave low, Passage horizontal, Passage vertical and Airlock 2.

## Discussion

Dark zone formation on wall surfaces of Lascaux’s Apse coincides with major shifts in microbial community composition [22], and the current work aimed at understanding the microbial similarities and particularities of dark zones in different locations of the cave in light of the presumed spatial heterogeneity of Lascaux Cave microbiome.

In a first stage, we clarified the level of spatial heterogeneity of the microbial community in Lascaux Cave. In various caves, microbiota heterogeneity has been documented in relation to (i) the distance to the cave entrance for Bacteria [50–52], Fungi [53] or Bacteria, Archaea and microeukaryotes together [12], (ii) different mineral surfaces e.g. weathered rock vs sediment (for Bacteria [51]), rock, sediment vs cave floor (for Bacteria and Fungi [23]), or various types of speleothems (for bacteria [54, 55]), and (iii) more contrasted types of environmental samples e.g. rock wall deposit, water sediment vs sinkhole soil (for bacteria [50]), or drip water, weathered rock, bat guano, sediment vs air (for fungi [53]). Here, NMDS and ANOSIM analyses showed that the microbial community on unmarked wall surfaces differed significantly across locations. The distance to the entrance might be a significant factor [56], with differences for Acidimicrobiia, Planctomycetes, Gammaproteobacteria, Eurotiomycetes, etc. between Airlock-2 wall (near the entrance) and the furthest areas e.g. Apse, Nave high and Nave low. However, climatic heterogeneity within the cave [56, 57] is likely limited by the existence of a door and several entry locks (Fig. 1B). A second key factor (largely confounding with the previous one) could be the type of limestone surface, with differences between natural cave limestone (e.g. higher relative abundance of Gammaproteobacteria), calcareous masonry benches (e.g. higher relative abundance of Alphaproteobacteria and Actinobacteria) and quarry limestone blocks (e.g. higher relative abundance of Chloroflexia and Planctomycetes). Mineral substrate particularities influence microbial communities [23, 54, 58]. A third factor is the application of chemical treatments aiming at curbing microbial outgrowth, which differed among locations in Lascaux Cave [6]. Perhaps the January 2008’ treatments in the Passage, Apse and Nave walls contributed to the higher abundance there of Gammaproteobacteria (in accordance with Bastian et al. [9]), as many of them are resistant to the chemicals used [59].

In a second stage, we compared dark zones and unmarked surfaces nearby. NMDS and ANOSIM analysis indicated that their microbiota differed at each of the nine locations. All dark zone alterations displayed counter-selection of the Gammaproteobacteria class, as already described in Lascaux Cave for the gammaproteobacterial genus *Pseudomonas* [22, 23]). Alphaproteobacteria selected on all dark zones (except on Passage vertical) included the Rhizobiales order, involved in alteration of paintings in Tomba Del Colle (Italy) [10]. It is interesting to note that dark zones started in the Apse in 2008 and formed at later times at locations closer to the cave entrance, with those on Airlock-2 wall appearing late 2016. Obviously, dark zone formation did not involve recent microbial invaders having made it into the cave via the entrance and modifying rock surface properties right away, and the occurrence of multiple taxa changes point to community-level modifications. *Folsomia candida* collembola have the potential to disseminate microorganisms [60, 61], but they are mainly observed in the Apse. Since microbiota variations associated with dark zones could differ from one location to the other, it suggests that changes in environmental conditions triggered these microbial dynamics. This might entail (i) long-term effects of previous chemical treatments, (ii) progressive changes in climatic conditions perhaps resulting from global warming and (since January 2015) the phasing-out of the air management system, or (iii) a combination of these factors. Further research will be needed to clarify this issue.

Many microorganisms may deteriorate materials by excretion of metabolites or extracellular enzymes [62], adhesion mechanisms or penetration of hyphae into microporous surfaces [7, 63–65]. This is likely to be promoted by mere microbial proliferation, and indeed qPCR levels of Bacteria and Fungi (but not of Archaea, which were strongly counter-selected) were significantly higher in dark zones than unmarked surfaces in all locations but on quarry limestone blocks of Airlock-2 wall and the masonry bench in Bulls left. Microbial proliferation is also indicated by direct, microscopy observations for the few samples of larger size that could be taken. Accordingly, the extensive use of chemical treatments also meant the introduction of organic carbon and nitrogen [6], and chemical analysis of cave floor samples did not point to N limitation [12].

In a third stage, we assessed the similarity of various dark zones to reference dark zones in the Apse, thanks to a decisional matrix based on criteria of the three domains of life stemming from previous observations [22]. They included the counter-selection of *Pseudomonas* and the selection of *Ochroconis*, which are important criteria for the reference dark zones of the Apse [21, 22]. On the basis of the decisional matrix, there was not a strong relationship with spatial distance/dark zone age or mineral surface, as distant masonry benches such as Bulls center and Bulls left (dark zones from 2016 on) displayed an overall similarity of respectively 40.0% and 37.4% with the Apse (where dark zones develop since 2008), whereas nearby natural surfaces of Nave high and Nave (dark zones from 2011 on) low had a similarity of 32.1% and 25.1%, respectively. Yet, the quarry limestone blocks in Airlock-2 wall and Bulls wall, also the most distant from the Apse and where dark zones were among the most recent ones (from 2016 and 2013, respectively), displayed only 24.0% and 20.8% similarity, respectively. Time might have been a meaningful factor influencing dark zone formation, provided the latter relied on complex microbial successions, but this may not be the case, at least in the Apse [22], and here correlations between (i) the date of dark zone appearance and (ii) Bray–Curtis distance between dark zone and control were not significant (even though for fungi it was not far off; *P* = 0.051). Similarity levels were not high overall (none exceeding the 40.2% value of Passage horizontal), especially since the contribution of individual criteria showing opposite dynamics were not accounted for. Therefore, in comparison with taxa associated with dark zone formation in the Apse, it suggests that key dark zone taxa at other Lascaux locations (i) may be endemic to these locations or at least present at higher population levels, and/or (ii) may correspond to cosmopolitan taxa but whose growth and contribution to alteration could be location-specific.

In a fourth stage, we assessed taxa based on biogeographic patterns. As many as 50 cosmopolitan genera were found, as well as 6 dark zone-specific genera found in all dark zone locations. The latter were only bacteria, including *Microbacterium* and *Bosea* described as producers of pigments such as carotenoids [66, 67]. The lack of dark zone-specific fungi found in all dark zone locations suggests that Bacteria may play a particular role in triggering dark zone formation. Perhaps dark zone formation involves also more location-specific contributions of other dark zone-specific microorganisms, i.e. (i) bacteria such as *Achromobacter* and *Nectria* in Nave high, *Hirschia* in Bulls wall, *Aliihoeflea* and *Arenibacter* in Bulls left, which may produce pigments [68–71], and (ii) Fungi e.g. *Bipolaris, Xylodon* and *Itersonilia* pointed in the Apse [22] and known for yellow–brown pigment production [72].

## Conclusions

Dark zone alterations are a major concern for Paleolithic art conservation in Lascaux, as they can form in various locations of the cave despite spatial heterogeneity of the microbial community. Microbiota differences between dark zones and unmarked surfaces at different locations present some similarities as well as location-specific properties. Microbial biogeographic patterns lead to the hypothesis that dark zone formation could entail proliferation of (i) a wide range of cosmopolitan bacterial and fungal taxa, (ii) six dark zone-specific bacterial genera found in all dark zone locations, and (iii) perhaps also other dark zone-specific Bacteria and Fungi found in certain but not all dark zone locations. Further work will target this issue. A better understanding of cave alteration dynamics may be useful to guide conservation strategies in Lascaux and other Paleolithic caves.

## Supplementary Information

**Additional file 1. Figure S1**: Correlation between dark zone age and Bray-Curtis distance of dark zone relatively to the control, for bacteria (A) and fungi (B). Dark zone age (in months) was the time elapsed since the appearance of dark zones in the Apse (i.e. June 2008). Kendall’s correlation coefficient and P value are indicated. **Figure S2**: Copy number values of microorganisms targeted by qPCR for each surface condition (n = 3). (A) Bacterial 16S rRNA gene, (B) Archaeal 16S rRNA gene and (C) Fungal ITS2 gene. **Table S1**: Primers for amplification of taxonomic marker genes. **Table S2**: Δlog Apse interval for score attribution for each criterion. **Table S3**: Adonis comparison of sampling location for bacterial 16S rRNA genes and for fungal ITS region, separately for control and dark zone conditions. **Table S4**: qPCR results (gene copies number/ng of total DNA) for bacterial, archaeal and fungal communities for control and dark zone conditions at each location in Lascaux Cave. **Table S5**: Pairwise-Adonis comparisons of sample locations for bacterial 16S rRNA genes and for fungal ITS region, separately for control and dark zone conditions. **Table S6**: Means and standard deviations (SD) of diversity indices (Simpson 1-D, Shannon H', Evenness index and Chao-1) for bacterial and fungal communities according to the rock surface condition and the sampling area. **Table S7**: Adonis comparison of rock surface conditions (control vs dark zone) and sample locations across the whole dataset for bacterial 16S rRNA genes and for fungal ITS region. **Table S8**: Pairwise-Adonis comparisons of control and dark zone conditions for bacterial 16S rRNA genes and for fungal ITS region, separately for each. **Table S9**: Computation of the 47 similarity scores for dark zones of Lascaux, in relation to the situation of the Apse's dark zones. For individual taxa, the raw data correspond to the mean number of sequences. SD, standard deviation (n = 6). Cell colors for scores correspond to the color code in Fig. 2. Scores without positive values are not indicated and the corresponding cell is displayed with a reddish color.

## Data Availability

All data that support findings of this study have been deposited in NCBI SRA database PRJNA798658, PRJNA799195 and PRJNA798756 for bacterial 16S rRNA genes, archaeal 16S rRNA genes and fungal ITS genes, respectively. The authors declare that the R (R 4.0.3) codes used to generate the results in this study are available in this paper. The R code supporting the finding presented here is available from the GitHub Repository https://github.com/LascauxZelia/Bontemps_et_al_DZ.

## Abbreviations

DNA: Deoxyribonucleic acid
PCR: Polymerase chain reaction
ITS: Internal transcribed spacer
OTU: Operational taxonomic unit
ANOVA: Analysis of variance
NMDS: Non-metric multi-dimensional scaling
qPCR: Quantitative polymerase chain reaction
ANOSIM: Analysis of similarities

## References

[1] Cigna A (2016). Tourism and show caves. Geomorphology.

[2] Brunet J, Vidal P, Vouvé J. The conservation of rock art. In: Studies and Documents on the Cultural Heritage. UNESCO;Paris.1987;7:1–17.

[3] Dupont J, Jacquet C, Dennetière B, Lacoste S, Bousta F, Orial G (2007). Invasion of the French Paleolithic painted cave of Lascaux by members of the *Fusarium solani* species complex. Mycologia.

[4] Russell MJ, MacLean VL (2008). Management issues in a Tasmanian tourist cave: Potential microclimatic impacts of cave modifications. J Environ Manag.

[5] Bastian F, Jurado V, Nováková A, Alabouvette C, Saiz-Jimenez C (2010). The microbiology of Lascaux Cave. Microbiology.

[6] Martin-Sanchez PM, Miller AZ, Saiz-Jimenez C. Lascaux Cave: an example of fragile ecological balance in subterranean environments. De Gruyter; Berlin/Boston. 2015; 279–301.

[7] Bontemps Z, Alonso L, Pommier T, Hugoni M, Moënne-Loccoz Y (2021). Microbial ecology of tourist Paleolithic caves. Sci Total Environ.

[8] Cañaveras JC, Sanchez-Moral S, Soler V, Saiz-Jimenez J (2001). Microorganisms and microbially induced fabrics in cave walls. Geomicrobiol J.

[9] Bastian F, Alabouvette C, Jurado V, Saiz-Jimenez C (2009). Impact of biocide treatments on the bacterial communities of the Lascaux Cave. Naturwissenschaften.

[10] Diaz-Herraiz M, Jurado V, Cuezva S, Laiz L, Pallecchi P, Tiano P (2014). Deterioration of an Etruscan tomb by bacteria from the order Rhizobiales. Sci Rep.

[11] Ikner LA, Toomey RS, Nolan G, Neilson JW, Pryor BM, Maier RM (2007). Culturable microbial diversity and the impact of tourism in Kartchner Caverns. Arizona Microb Ecol.

[12] Alonso L, Pommier T, Kaufmann B, Dubost A, Chapulliot D, Doré J (2019). Anthropization level of Lascaux Cave microbiome shown by regional-scale comparisons of pristine and anthropized caves. Mol Ecol.

[13] He J, Zhang N, Muhammad A, Shen X, Sun C, Li Q (2022). From surviving to thriving, the assembly processes of microbial communities in stone biodeterioration: a case study of the West Lake UNESCO World Heritage area in China. Sci Total Environ.

[14] La LM (1974). ‘Maladie Verte’ de Lascaux. Stud Conserv.

[15] Bastian F, Alabouvette C (2009). Lights and shadows on the conservation of a rock art cave: the case of Lascaux Cave. Int J Speleol.

[16] Baquedano Estévez C, Merino LM, de la Losa Román A, Valsero JD (2019). The lampenflora in show caves and its treatment: an emerging ecological problem. Int J Speleol.

[17] Chalmin E, d’Orlyé F, Zinger L, Charlet L, Geremia RA, Orial G (2007). Biotic versus abiotic calcite formation on prehistoric cave paintings: the Arcy-sur-Cure ‘Grande Grotte’ (Yonne, France) case. Geol Soc London.

[18] Portillo MC, Gonzalez JM, Saiz-Jimenez C (2008). Metabolically active microbial communities of yellow and grey colonizations on the walls of Altamira Cave, Spain. J Appl Microbiol.

[19] Portillo MC, Gonzalez JM (2010). Differential effects of distinct bacterial biofilms in a cave environment. Curr Microbiol.

[20] Urzì C, De Leo F, Bruno L, Albertano P (2010). Microbial diversity in Paleolithic caves: a study case on the phototrophic biofilms of the Cave of Bats (Zuheros, Spain). Microb Ecol.

[21] Alonso L, Pommier T, Abrouk D, Hugoni M, Tran VT, Minard G (2022). Microbiome analysis of new, insidious cave wall alterations in the Apse of Lascaux Cave. Microorganisms.

[22] Bontemps Z, Hugoni M, Moënne-Loccoz Y (2023). Microscale dynamics of dark zone alterations in anthropized karstic cave shows abrupt microbial community switch. Sci Total Environ.

[23] Alonso L, Creuzé-des-Châtelliers C, Trabac T, Dubost A, Moënne-Loccoz Y, Pommier T (2018). Rock substrate rather than black stain alterations drives microbial community structure in the passage of Lascaux Cave. Microbiome.

[24] Herlemann DP, Labrenz M, Jürgens K, Bertilsson S, Waniek JJ, Andersson AF (2011). Transitions in bacterial communities along the 2000 km salinity gradient of the Baltic Sea. ISME J.

[25] Herfort L, Kim J-H, Coolen MJL, Abbas B, Schouten S, Herndl GJ (2009). Diversity of Archaea and detection of crenarchaeotal *amoA* genes in the rivers Rhine and Têt. Aquat Microb Ecol.

[26] Toju H, Tanabe AS, Yamamoto S, Sato H (2021). High-Coverage ITS primers for the DNA-based identification of Ascomycetes and Basidiomycetes in environmental samples. PLoS ONE.

[27] Magoč T, Salzberg SL (2011). FLASH: fast length adjustment of short reads to improve genome assemblies. Bioinformatics Oxf Engl.

[28] Mahé F, Rognes T, Quince C, de Vargas C, Dunthorn M (2014). Swarm: robust and fast clustering method for amplicon-based studies. PeerJ.

[29] Rognes T, Flouri T, Nichols B, Quince C, Mahé F (2016). VSEARCH: a versatile open source tool for metagenomics. PeerJ.

[30] Bokulich NA, Subramanian S, Faith JJ, Gevers D, Gordon JI, Knight R (2013). Quality-filtering vastly improves diversity estimates from Illumina amplicon sequencing. Nat Methods.

[31] Zhang J, Madden TL (1997). PowerBLAST: a new network BLAST application for interactive or automated sequence analysis and annotation. Genome Res.

[32] Quast C, Pruesse E, Yilmaz P, Gerken J, Schweer T, Yarza P (2013). The SILVA ribosomal RNA gene database project: improved data processing and web-based tools. Nucleic Acids Res.

[33] Nilsson RH, Larsson K-H, Taylor AFS, Bengtsson-Palme J, Jeppesen TS, Schigel D (2019). The UNITE database for molecular identification of fungi: handling dark taxa and parallel taxonomic classifications. Nucleic Acids Res.

[34] Escudié F, Auer L, Bernard M, Mariadassou M, Cauquil L, Vidal K (2018). FROGS: find, rapidly, OTUs with galaxy solution. Bioinformatics Oxf Engl.

[35] Chao A (1987). Estimating the population size for capture-recapture data with unequal catchability. Biometrics.

[36] Shannon CE (1948). A mathematical theory of communication. Bell Syst Tech J.

[37] Simpson EH (1949). Measurement of diversity. Nature.

[38] Hammer Ø, Harper D, Ryan P (2001). PAST: paleontological statistics software package for education and data analysis. Palaeontol Electron.

[39] Oksanen J, Blanchet FG, Friendly M, Kindt R, Legendre P, McGlinn D, et al. vegan: community ecology package. 2020. Available from: https://CRAN.R-project.org/package=vegan.

[40] McMurdie PJ, Holmes S (2013). phyloseq: an R package for reproducible interactive analysis and graphics of microbiome census data. PLoS ONE.

[41] Kassambara A, Mundt F. factoextra: extract and visualize the results of multivariate data analyses. 2020. Available from: https://CRAN.R-project.org/package=factoextra.

[42] Husson F, Josse J, Le S, Mazet J. FactoMineR: multivariate exploratory data analysis and data mining. 2020. Available from: https://CRAN.R-project.org/package=FactoMineR.

[43] Wickham H, François R, Henry L, Müller K, RStudio. dplyr: a grammar of data manipulation. 2021. Available from: https://CRAN.R-project.org/package=dplyr.

[44] Clarke KR (1993). Non-parametric multivariate analyses of changes in community structure. Aust J Ecol.

[45] R Core Team. R: A language and environment for statistical computing. Vienna, Austria: R Foundation for Statistical Computing; 2020. Available from: https://www.R-project.org/.

[46] Lane DJ. 16S/23S rRNA sequencing. In: Stackebrandt E, Goodfellow M, editors. Nucleic Acid Techniques in Bacterial Systematics. Chichester, England: Wiley; 1991. p. 205–248.

[47] Nehmé B, Gilbert Y, Létourneau V, Forster RJ, Veillette M, Villemur R (2009). Culture-independent characterization of archaeal biodiversity in swine confinement building bioaerosols. Appl Environ Microbiol.

[48] Hugoni M, Luis P, Guyonnet J, Haichar F (2018). Plant host habitat and root exudates shape fungal diversity. Mycorrhiza.

[49] Van Rossum G, Drake F. Python 3 reference manual. Scotts Valley: CA: CreateSpace; 2009.

[50] Wu Y, Tan L, Liu W, Wang B, Wang J, Cai Y (2015). Profiling bacterial diversity in a limestone cave of the western Loess Plateau of China. Front Microbiol.

[51] Ma L, Huang X, Wang H, Yun Y, Cheng X, Liu D (2021). Microbial interactions drive distinct taxonomic and potential metabolic responses to habitats in karst cave ecosystem. Microbiol Spectr.

[52] Buresova-Faitova A, Kopecky J, Sagova-Mareckova M, Alonso L, Vautrin F, Moënne-Loccoz Y (2022). Comparison of Actinobacteria communities from human-impacted and pristine karst caves. MicrobiologyOpen.

[53] Man B, Wang H, Yun Y, Xiang X, Wang R, Duan Y (2018). Diversity of fungal communities in Heshang Cave of central China revealed by mycobiome-sequencing. Front Microbiol.

[54] Dhami NK, Mukherjee A, Watkin ELJ (2018). Microbial diversity and mineralogical-mechanical properties of calcitic cave speleothems in natural and in vitro biomineralization conditions. Front Microbiol.

[55] Tok E, Olgun N, Dalfes HN (2021). Profiling bacterial diversity in relation to different habitat types in a limestone cave: İnsuyu Cave. Turkey Geomicrobiol J.

[56] Bourges F, Genthon P, Genty D, Lorblanchet M, Mauduit E, D’Hulst D (2014). Conservation of prehistoric caves and stability of their inner climate: Lessons from Chauvet and other French caves. Sci Total Environ.

[57] Lacanette D, Vincent S, Sarthou A, Malaurent P, Caltagirone J-P (2009). An Eulerian/Lagrangian method for the numerical simulation of incompressible convection flows interacting with complex obstacles: application to the natural convection in the Lascaux cave. Int J Heat Mass Transf.

[58] Brewer TE, Fierer N (2018). Tales from the tomb: the microbial ecology of exposed rock surfaces. Environ Microbiol.

[59] Merchel Piovesan Pereira B, Tagkopoulos I (2019). Benzalkonium chlorides: Uses, regulatory status, and microbial resistance. Appl Environ Microbiol.

[60] Bastian F, Alabouvette C, Saiz-Jimenez C (2009). The impact of arthropods on fungal community structure in Lascaux Cave. J Appl Microbiol.

[61] Alonso L, Pommier T, Simon L, Maucourt F, Doré J, Dubost A (2022). Microbiome analysis in Lascaux Cave in relation to black stain alterations of rock surfaces and collembola. Environ Microbiol Rep.

[62] Sand W (1997). Microbial mechanisms of deterioration of inorganic substrates: a general mechanistic overview. Int Biodeterior Biodegrad.

[63] Riding R (2000). Microbial carbonates: the geological record of calcified bacterial–algal mats and biofilms. Sedimentology.

[64] Gorbushina AA (2007). Life on the rocks. Environ Microbiol.

[65] Zucconi L, Gagliardi M, Isola D, Onofri S, Andaloro MC, Pelosi C (2012). Biodeterioration agents dwelling in or on the wall paintings of the Holy Saviour’s cave (Vallerano, Italy). Int Biodeterior Biodegrad.

[66] Meddeb-Mouelhi F, Moisan JK, Bergeron J, Daoust B, Beauregard M (2016). Structural characterization of a novel antioxidant pigment produced by a photochromogenic *Microbacterium oxydans* strain. Appl Biochem Biotechnol.

[67] Albert RA, McGuine M, Pavlons SC, Roecker J, Bruess J, Mossman S (2019). *Bosea psychrotolerans* sp. Nov., a psychrotrophic alphaproteobacterium isolated from Lake Michigan water. Int J Syst Evol Microbiol.

[68] Ivanova V, Tomova I, Kamburov A, Tomova A, Vasileva-Tonkova E, Kambourova M (2013). High phylogenetic diversity of bacteria in the area of prehistoric paintings in Magura Cave. Bulgaria J Cave Karst Stud.

[69] Kang HS, Lee SD (2009). *Hirschia maritima* sp. Nov., isolated from seawater. Int J Syst Evol Microbiol.

[70] Parisot D, Devys M, Férézou J-P, Barbier M (1983). Pigments from *Nectria haematococca*: Anhydrofusarubin lactone and nectriafurone. Phytochemistry.

[71] Duerre JA, Buckley PJ (1965). Pigment production from tryptophan by an *Achromobacter* species. J Bacteriol.

[72] Sharma P, Gi̇ll PK, Aggarwal A (2014). *Bipolaris spicifera*, an unusual cause of non-healing cutaneous ulcer in an immunocompromised patient. J Microbiol Infect Dis.

