# Supplementary material for: Dark-zone alterations expand throughout Paleolithic Lascaux Cave despite spatial heterogeneity of the cave microbiome
Source: Environ Microbiome. 2023 Apr 10;18:31. doi: 10.1186/s40793-023-00488-8 (PMC10084675; doi:10.1186/s40793-023-00488-8)
Supplement: Supplementary file 1 — Additional file 1. Figure S1: Correlation between dark zone age and Bray-Curtis distance of dark zone relatively to the control, for bacteria (A) and fungi (B). Dark zone age (in months) was the time elapsed since the appearance of dark zones in the Apse (i.e. June 2008). Kendall’s correlation coefficient and P value are indicated. Figure S2: Copy number values of microorganisms targeted by qPCR for each surface condition (n = 3). (A) Bacterial 16S rRNA gene, (B) Archaeal 16S rRNA gene and (C) Fungal ITS2 gene. Table S1: Primers for amplification of taxonomic marker genes. Table S2: Δlog Apse interval for score attribution for each criterion. Table S3: Adonis comparison of sampling location for bacterial 16S rRNA genes and for fungal ITS region, separately for control and dark zone conditions. Table S4: qPCR results (gene copies number/ng of total DNA) for bacterial, archaeal and fungal communities for control and dark zone conditions at each location in Lascaux Cave. Table S5: Pairwise-Adonis comparisons of sample locations for bacterial 16S rRNA genes and for fungal ITS region, separately for control and dark zone conditions. Table S6: Means and standard deviations (SD) of diversity indices (Simpson 1-D, Shannon H', Evenness index and Chao-1) for bacterial and fungal communities according to the rock surface condition and the sampling area. Table S7: Adonis comparison of rock surface conditions (control vs dark zone) and sample locations across the whole dataset for bacterial 16S rRNA genes and for fungal ITS region. Table S8: Pairwise-Adonis comparisons of control and dark zone conditions for bacterial 16S rRNA genes and for fungal ITS region, separately for each. Table S9: Computation of the 47 similarity scores for dark zones of Lascaux, in relation to the situation of the Apse's dark zones. For individual taxa, the raw data correspond to the mean number of sequences. SD, standard deviation (n = 6). Cell colors for scores correspond to the color code in Fig. 2. [file 40793_2023_488_MOESM1_ESM.pdf]

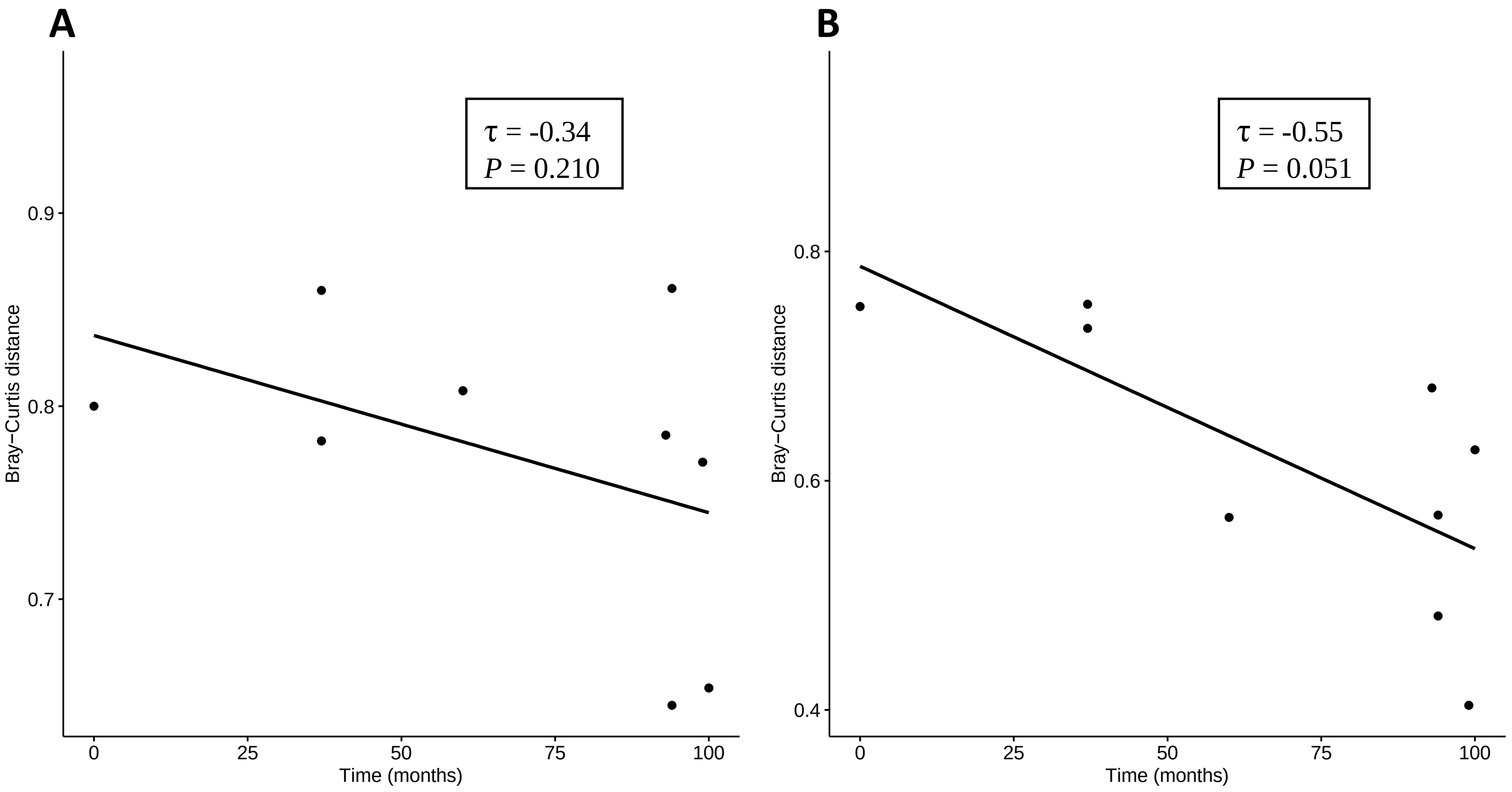
A
B
τ = -0.34
P = 0.210
τ = -0.55
P = 0.051
Bray−Curtis distance
Time (months)
0.9
0.8
0.7
0.6
0.4
0
25
50
75
100

**Fig S1**

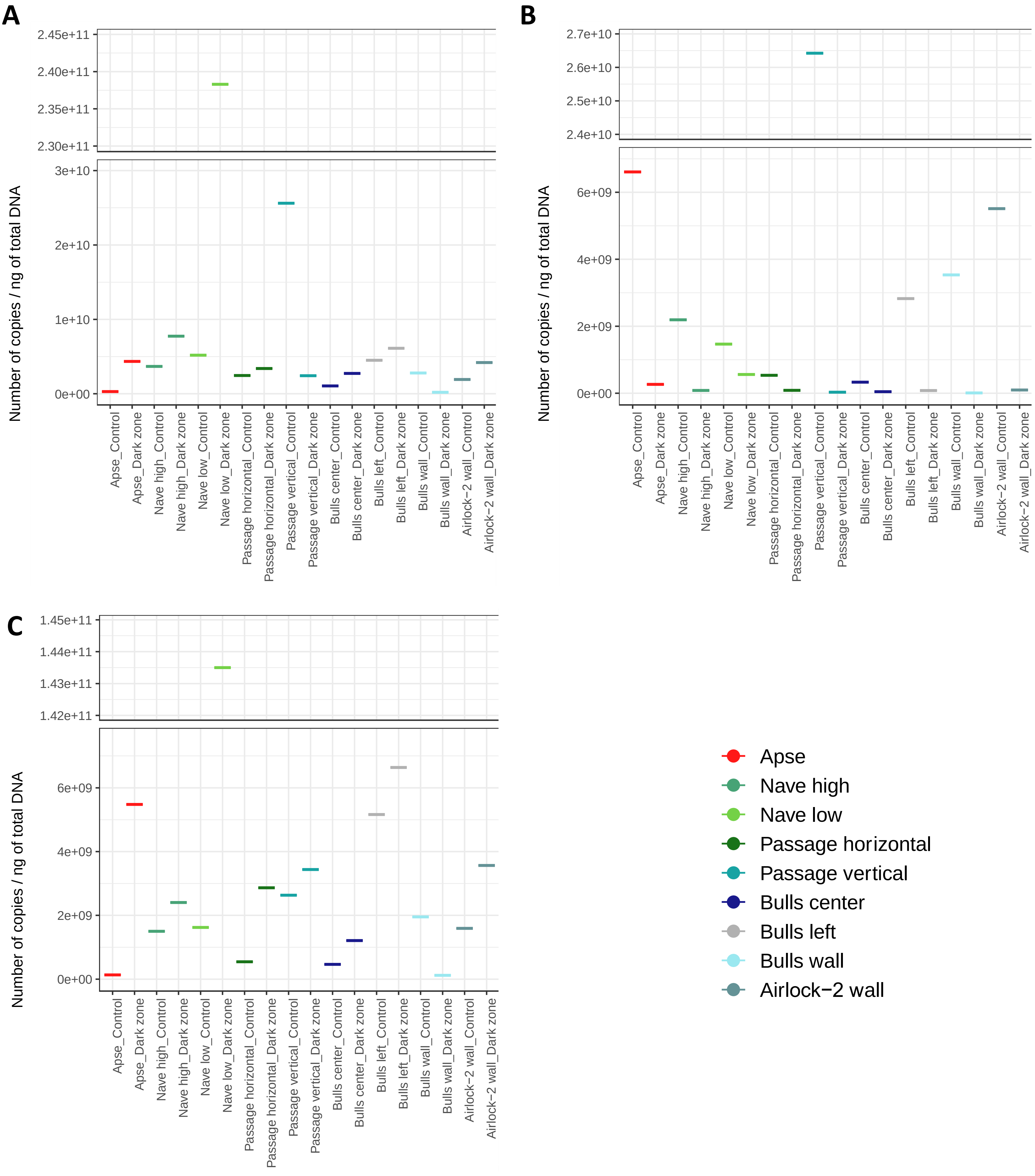

A
B
C
Number of copies / ng of total DNA
2.45e+11
2.40e+11
2.35e+11
2.30e+11
3e+10
2e+10
1e+10
0e+00
2.7e+10
2.6e+10
2.5e+10
2.4e+10
6e+09
4e+09
2e+09
0e+00
1.45e+11
1.44e+11
1.43e+11
1.42e+11
Apse_Control
Apse_Dark zone
Nave high_Control
Nave high_Dark zone
Nave low_Control
Nave low_Dark zone
Passage horizontal_Control
Passage horizontal_Dark zone
Passage vertical_Control
Passage vertical_Dark zone
Bulls center_Control
Bulls center_Dark zone
Bulls left_Control
Bulls left_Dark zone
Bulls wall_Control
Bulls wall_Dark zone
Airlock−2 wall_Control
Airlock−2 wall_Dark zone
Apse
Nave high
Nave low
Passage horizontal
Passage vertical
Bulls center
Bulls left
Bulls wall
Airlock−2 wall


**Fig S2**

**Table S1.** Primers for amplification of taxonomic marker genes for metabarcoding.

| **Marker gene** | **Region** | **Length** | **Name** | **Forward (F) or reverse (R)** | **Sequence** | **Reference** |
|---|---|---|---|---|---|---|
| 16S rRNA | V3-V4 Bacteria | 550 bp | 341F | F | 5'-CCTACGGGNGGCWGCAG-3' | Herlemann et al. 2011 |
| | | | 805R | R | 5'-GACTACHVGGGTATCTAATCC-3' | |
| | V3-V4 Archaea | 420 bp | 515F | F | 5'-CAGCCGCCGCGGTAA-3' | Herfort et al. 2011 |
| | | | 915R | R | 5'-GTGCTCCCCCGCCAATTCCT-3’ | |
| ITS | ITS2 | 327 bp | ITS3_KYO2 | F | 5'-GATGAAGAACGYAGYRAA-3’ | Toju et al. 2012 |
| | | | ITS4 | R | 5'-TCCTCCGCTTATTGATATGC- 3’ | |

Herfort L, Kim J-H, Coolen MJL, Abbas B, Schouten S, Herndl GJ, et al. Diversity of Archaea and detection of crenarchaeotal *amoA* genes in the rivers Rhine and Têt. Aquat Microb Ecol. 2009;55:189–201.
Herlemann DP, Labrenz M, Jürgens K, Bertilsson S, Waniek JJ, Andersson AF. Transitions in bacterial communities along the 2000 km salinity gradient of the Baltic Sea. ISME J. 2011;5:1571–9.
Toju H, Tanabe AS, Yamamoto S, Sato H. High-Coverage ITS primers for the DNA-based identification of Ascomycetes and Basidiomycetes in environmental samples. PLoS ONE. 2021;7:11-8.

**Supplementary Table S2.** Δlog Apse interval for score attribution for each criterion.

| Criteria | Δlog Apse + 1 Δlog Apse | Δlog Apse + 0.9Δlog Apse | Δlog Apse + 0.8Δlog Apse | Δlog Apse + 0.7Δlog Apse | Δlog Apse + 0.6Δlog Apse | Δlog Apse + 0.5Δlog Apse | Δlog Apse + 0.4Δlog Apse | Δlog Apse + 0.3Δlog Apse | Δlog Apse + 0.2Δlog Apse | Δlog Apse + 0.1Δlog Apse | Δlog Apse | Δlog Apse - 0.1Δlog Apse | Δlog Apse - 0.2Δlog Apse | Δlog Apse - 0.3Δlog Apse | Δlog Apse - 0.4Δlog Apse | Δlog Apse - 0.5Δlog Apse | Δlog Apse - 0.6Δlog Apse | Δlog Apse - 0.7Δlog Apse | Δlog Apse - 0.8Δlog Apse | Δlog Apse - 0.9Δlog Apse | Δlog Apse - 1 Δlog Apse |
|---|---|---|---|---|---|---|---|---|---|---|---|---|---|---|---|---|---|---|---|---|---|
| *Niabella* | 5.781 | 5.492 | 5.203 | 4.914 | 4.625 | 4.336 | 4.047 | 3.758 | 3.469 | 3.180 | 2.890 | 2.601 | 2.312 | 2.023 | 1.734 | 1.445 | 1.156 | 0.867 | 0.578 | 0.289 | 0.000 |
| *Steroidobacter* | 4.824 | 4.583 | 4.342 | 4.100 | 3.859 | 3.618 | 3.377 | 3.135 | 2.894 | 2.653 | 2.412 | 2.171 | 1.929 | 1.688 | 1.447 | 1.206 | 0.964 | 0.723 | 0.482 | 0.241 | 0.000 |
| *Acidothermus* | 4.629 | 4.398 | 4.166 | 3.935 | 3.703 | 3.472 | 3.240 | 3.009 | 2.777 | 2.546 | 2.314 | 2.083 | 1.851 | 1.620 | 1.388 | 1.157 | 0.925 | 0.694 | 0.462 | 0.231 | 0.000 |
| *Phaselicystis* | 4.454 | 4.231 | 4.008 | 3.785 | 3.563 | 3.340 | 3.117 | 2.895 | 2.672 | 2.449 | 2.227 | 2.004 | 1.781 | 1.558 | 1.336 | 1.113 | 0.890 | 0.668 | 0.445 | 0.222 | 0.000 |
| *Saccharopolyspora* | 4.171 | 3.963 | 3.754 | 3.546 | 3.337 | 3.128 | 2.920 | 2.711 | 2.503 | 2.294 | 2.085 | 1.877 | 1.668 | 1.460 | 1.251 | 1.042 | 0.834 | 0.625 | 0.417 | 0.208 | 0.000 |
| *Nitrospira* | 4.139 | 3.932 | 3.725 | 3.518 | 3.311 | 3.104 | 2.897 | 2.690 | 2.483 | 2.276 | 2.069 | 1.862 | 1.655 | 1.448 | 1.241 | 1.034 | 0.827 | 0.620 | 0.413 | 0.206 | 0.000 |
| *Jatrophihabitans* | 3.480 | 3.306 | 3.132 | 2.958 | 2.784 | 2.610 | 2.436 | 2.262 | 2.088 | 1.914 | 1.740 | 1.566 | 1.392 | 1.218 | 1.044 | 0.870 | 0.696 | 0.522 | 0.348 | 0.174 | 0.000 |
| *Nonomuraea* | 3.305 | 3.140 | 2.975 | 2.809 | 2.644 | 2.479 | 2.3144 | 2.148 | 1.983 | 1.818 | 1.652 | 1.487 | 1.322 | 1.157 | 0.991 | 0.826 | 0.661 | 0.495 | 0.330 | 0.165 | 0.000 |
| *Tundrisphaera* | 3.296 | 3.131 | 2.966 | 2.802 | 2.637 | 2.472 | 2.307 | 2.142 | 1.977 | 1.812 | 1.648 | 1.483 | 1.318 | 1.153 | 0.988 | 0.824 | 0.659 | 0.494 | 0.329 | 0.164 | 0.000 |
| *Bryobacter* | 3.267 | 3.104 | 2.941 | 2.777 | 2.614 | 2.450 | 2.287 | 2.124 | 1.960 | 1.797 | 1.633 | 1.470 | 1.307 | 1.143 | 0.980 | 0.816 | 0.653 | 0.490 | 0.326 | 0.163 | 0.000 |
| *Flavihumibacter* | 3.175 | 3.016 | 2.857 | 2.699 | 2.540 | 2.381 | 2.222 | 2.064 | 1.905 | 1.746 | 1.587 | 1.428 | 1.270 | 1.111 | 0.952 | 0.793 | 0.635 | 0.476 | 0.317 | 0.158 | 0.000 |
| *Actinophytocola* | 3.069 | 2.916 | 2.762 | 2.609 | 2.455 | 2.302 | 2.148 | 1.995 | 1.841 | 1.688 | 1.534 | 1.381 | 1.227 | 1.074 | 0.920 | 0.767 | 0.613 | 0.460 | 0.306 | 0.153 | 0.000 |
| *Alysiosphaera* | 2.996 | 2.846 | 2.696 | 2.547 | 2.397 | 2.247 | 2.097 | 1.947 | 1.797 | 1.648 | 1.498 | 1.348 | 1.198 | 1.048 | 0.898 | 0.749 | 0.599 | 0.449 | 0.299 | 0.149 | 0.000 |
| *Galbitalea* | 2.982 | 2.833 | 2.684 | 2.535 | 2.386 | 2.237 | 2.087 | 1.938 | 1.789 | 1.640 | 1.491 | 1.342 | 1.193 | 1.043 | 0.894 | 0.745 | 0.596 | 0.447 | 0.298 | 0.149 | 0.000 |
| *Microlunatus* | 2.878 | 2.734 | 2.590 | 2.446 | 2.302 | 2.158 | 2.015 | 1.871 | 1.727 | 1.583 | 1.439 | 1.295 | 1.151 | 1.007 | 0.863 | 0.719 | 0.575 | 0.431 | 0.287 | 0.143 | 0.000 |
| Unidentified (Solirubrobacterales) | 2.683 | 2.549 | 2.415 | 2.280 | 2.146 | 2.012 | 1.878 | 1.744 | 1.610 | 1.475 | 1.341 | 1.207 | 1.073 | 0.939 | 0.805 | 0.670 | 0.536 | 0.402 | 0.268 | 0.134 | 0.000 |
| Unidentified (Thermomicrobiales) | 2.373 | 2.255 | 2.136 | 2.017 | 1.899 | 1.780 | 1.661 | 1.543 | 1.424 | 1.305 | 1.186 | 1.068 | 0.949 | 0.830 | 0.712 | 0.593 | 0.474 | 0.356 | 0.237 | 0.118 | 0.000 |
| *Jiangella* | 2.212 | 2.102 | 1.991 | 1.880 | 1.770 | 1.659 | 1.549 | 1.438 | 1.327 | 1.217 | 1.106 | 0.995 | 0.885 | 0.774 | 0.663 | 0.553 | 0.442 | 0.331 | 0.221 | 0.110 | 0.000 |
| *Sphingopyxis* | 2.109 | 2.003 | 1.898 | 1.792 | 1.687 | 1.581 | 1.476 | 1.371 | 1.265 | 1.160 | 1.054 | 0.949 | 0.843 | 0.738 | 0.632 | 0.527 | 0.421 | 0.316 | 0.210 | 0.105 | 0.000 |
| *Afipia* | 2.059 | 1.956 | 1.853 | 1.750 | 1.647 | 1.544 | 1.441 | 1.338 | 1.235 | 1.132 | 1.029 | 0.926 | 0.823 | 0.720 | 0.617 | 0.514 | 0.411 | 0.308 | 0.205 | 0.102 | 0.000 |
| *Phenylobacterium* | 2.017 | 1.916 | 1.815 | 1.714 | 1.613 | 1.512 | 1.412 | 1.311 | 1.210 | 1.109 | 1.008 | 0.907 | 0.806 | 0.706 | 0.605 | 0.504 | 0.403 | 0.302 | 0.201 | 0.100 | 0.000 |
| *Methylopila* | -2.271 | -2.157 | -2.044 | -1.930 | -1.817 | -1.703 | -1.589 | -1.476 | -1.362 | -1.249 | -1.135 | -1.022 | -0.908 | -0.794 | -0.681 | -0.567 | -0.454 | -0.340 | -0.227 | -0.113 | 0.000 |
| *Streptomyces* | -2.492 | -2.367 | -2.242 | -2.118 | -1.993 | -1.869 | -1.744 | -1.619 | -1.495 | -1.370 | -1.246 | -1.121 | -0.996 | -0.872 | -0.747 | -0.623 | -0.498 | -0.373 | -0.249 | -0.124 | 0.000 |
| *Reyranella* | -2.574 | -2.445 | -2.316 | -2.188 | -2.059 | -1.930 | -1.801 | -1.673 | -1.544 | -1.415 | -1.287 | -1.158 | -1.029 | -0.900 | -0.772 | -0.643 | -0.514 | -0.386 | -0.257 | -0.128 | 0.000 |
| *Rhodopseudomonas* | -3.625 | -3.444 | -3.263 | -3.081 | -2.900 | -2.719 | -2.538 | -2.356 | -2.175 | -1.994 | -1.812 | -1.631 | -1.450 | -1.269 | -1.087 | -0.906 | -0.725 | -0.543 | -0.362 | -0.181 | 0.000 |
| Unidentified (Alcaligenaceae) | -4.408 | -4.188 | -3.967 | -3.747 | -3.526 | -3.306 | -3.085 | -2.865 | -2.645 | -2.424 | -2.204 | -1.983 | -1.763 | -1.542 | -1.322 | -1.102 | -0.881 | -0.661 | -0.440 | -0.220 | 0.000 |
| *Pseudomonas* | -6.187 | -5.877 | -5.568 | -5.259 | -4.949 | -4.640 | -4.331 | -4.021 | -3.712 | -3.403 | -3.093 | -2.784 | -2.474 | -2.165 | -1.856 | -1.546 | -1.237 | -0.928 | -0.618 | -0.309 | 0.000 |
| 16S Copy number / ng DNA | 2.342 | 2.225 | 2.108 | 1.991 | 1.873 | 1.756 | 1.639 | 1.522 | 1.405 | 1.288 | 1.171 | 1.054 | 0.936 | 0.819 | 0.702 | 0.585 | 0.468 | 0.351 | 0.234 | 0.117 | 0.000 |
| Number of reads | -2.076 | -1.971 | -1.868 | -1.764 | -1.660 | -1.550 | -1.453 | -1.349 | -1.245 | -1.141 | -1.037 | -0.934 | -0.830 | -0.726 | -0.622 | -0.518 | -0.415 | -0.311 | -0.207 | -0.103 | 0.000 |
| 16S Copy number / ng DNA | -5.722 | -5.436 | -5.150 | -4.863 | -4.577 | -4.291 | -4.005 | -3.719 | -3.433 | -3.147 | -2.861 | -2.575 | -2.288 | -2.002 | -1.716 | -1.430 | -1.144 | -0.858 | -0.572 | -0.286 | 0.000 |
| *Chrysosporium* | -6.851 | -6.509 | -6.166 | -5.823 | -5.481 | -5.138 | -4.796 | -4.453 | -4.111 | -3.768 | -3.425 | -3.083 | -2.740 | -2.398 | -2.055 | -1.712 | -1.370 | -1.027 | -0.685 | -0.342 | 0.000 |
| *Pseudogymnoascus* | -5.974 | -5.676 | -5.377 | -5.078 | -4.779 | -4.481 | -4.182 | -3.883 | -3.584 | -3.286 | -2.987 | -2.688 | -2.389 | -2.091 | -1.792 | -1.493 | -1.194 | -0.896 | -0.597 | -0.298 | 0.000 |
| *Illyonectria* | -5.288 | -5.024 | -4.759 | -4.495 | -4.231 | -3.966 | -3.702 | -3.437 | -3.173 | -2.908 | -2.644 | -2.379 | -2.115 | -1.851 | -1.586 | -1.322 | -1.057 | -0.793 | -0.528 | -0.264 | 0.000 |
| *Trichoderma* | -4.949 | -4.702 | -4.454 | -4.207 | -3.959 | -3.712 | -3.464 | -3.217 | -2.969 | -2.722 | -2.474 | -2.227 | -1.979 | -1.732 | -1.484 | -1.237 | -0.989 | -0.742 | -0.494 | -0.247 | 0.000 |
| *Serendipita* | -4.130 | -3.924 | -3.717 | -3.510 | -3.304 | -3.097 | -2.891 | -2.684 | -2.478 | -2.271 | -2.065 | -1.858 | -1.652 | -1.445 | -1.239 | -1.032 | -0.826 | -0.619 | -0.413 | -0.206 | 0.000 |
| *Cephalotrichum* | -3.899 | -3.703 | -3.508 | -3.313 | -3.119 | -2.924 | -2.729 | -2.534 | -2.339 | -2.144 | -1.949 | -1.754 | -1.559 | -1.364 | -1.169 | -0.974 | -0.779 | -0.584 | -0.389 | -0.194 | 0.000 |
| Unidentified (Hypocreales) | -3.241 | -3.079 | -2.917 | -2.755 | -2.593 | -2.431 | -2.269 | -2.107 | -1.945 | -1.782 | -1.620 | -1.458 | -1.296 | -1.134 | -0.972 | -0.810 | -0.648 | -0.486 | -0.324 | -0.162 | 0.000 |
| *Oidodendron* | -2.650 | -2.517 | -2.385 | -2.252 | -2.120 | -1.987 | -1.855 | -1.722 | -1.590 | -1.457 | -1.325 | -1.192 | -1.060 | -0.927 | -0.795 | -0.662 | -0.530 | -0.397 | -0.265 | -0.132 | 0.000 |
| Unidentified | -2.370 | -2.252 | -2.133 | -2.015 | -1.896 | -1.778 | -1.659 | -1.541 | -1.422 | -1.304 | -1.185 | -1.066 | -0.948 | -0.829 | -0.711 | -0.592 | -0.474 | -0.355 | -0.237 | -0.118 | 0.000 |
| *Clonostachys* | -2.101 | -1.996 | -1.891 | -1.785 | -1.680 | -1.575 | -1.470 | -1.365 | -1.260 | -1.155 | -1.050 | -0.945 | -0.840 | -0.735 | -0.630 | -0.525 | -0.420 | -0.315 | -0.210 | -0.105 | 0.000 |
| *Gliomastix* | -2.024 | -1.923 | -1.822 | -1.720 | -1.619 | -1.518 | -1.417 | -1.315 | -1.214 | -1.113 | -1.012 | -0.911 | -0.809 | -0.708 | -0.607 | -0.506 | -0.404 | -0.303 | -0.202 | -0.101 | 0.000 |
| *Ochroconis* | 2.225 | 2.113 | 2.002 | 1.891 | 1.780 | 1.668 | 1.557 | 1.446 | 1.335 | 1.223 | 1.112 | 1.001 | 0.890 | 0.778 | 0.667 | 0.556 | 0.445 | 0.333 | 0.222 | 0.111 | 0.000 |
| *Mortierella* | 4.437 | 4.215 | 3.993 | 3.771 | 3.549 | 3.328 | 3.106 | 2.884 | 2.662 | 2.440 | 2.218 | 1.996 | 1.774 | 1.553 | 1.331 | 1.109 | 0.887 | 0.665 | 0.443 | 0.221 | 0.000 |
| *Saccharomyces* | 4.652 | 4.420 | 4.187 | 3.954 | 3.722 | 3.489 | 3.256 | 3.024 | 2.791 | 2.558 | 2.326 | 2.093 | 1.861 | 1.628 | 1.395 | 1.163 | 0.930 | 0.697 | 0.465 | 0.232 | 0.000 |
| ITS Copy number / ng DNA | 3.230 | 3.068 | 2.907 | 2.745 | 2.584 | 2.422 | 2.261 | 2.099 | 1.938 | 1.776 | 1.615 | 1.453 | 1.292 | 1.130 | 0.969 | 0.807 | 0.640 | 0.484 | 0.323 | 0.161 | 0.000 |

**Table S3.** Adonis comparison of sampling location for bacterial 16S rRNA genes and for fungal ITS region, separately for control and dark zone conditions.

| | | **Bacterial 16S rRNA gene** | | | | **Fungal ITS region** | | | |
|---|---|---|---|---|---|---|---|---|---|
| | | **Df** | **F** | ***P*** | $\mathbf{R^2}$ | **Df** | **F** | ***P*** | $\mathbf{R^2}$ |
| Control | *Effect :* | | | | | | | | |
| | Location | 8 | 12.835 | **0.001** | 0.695 | 8 | 9.338 | **0.007** | 0.629 |
| | Residuals | 53 | | | | 53 | | | |
| Dark zone | *Effect :* | | | | | | | | |
| | Location | 8 | 9.784 | **0.008** | 0.634 | 8 | 7.005 | **0.010** | 0.560 |
| | Residuals | 53 | | | | 53 | | | |

**Table S4**. qPCR results (number of gene copies / ng of total DNA) for bacterial, archaeal and fungal communities for control and dark zone conditions at each location in Lascaux Cave. SD, Standard deviation.

| | **Bacteria 16S rRNA** | | | | **Archaea 16S rRNA** | | | | **Fungi ITS** | | | |
|---|---|---|---|---|---|---|---|---|---|---|---|---|
| | Control | | Dark zone | | Control | | Dark zone | | Control | | Dark zone | |
| Sampling location | Mean | SD | Mean | SD | Mean | SD | Mean | SD | Mean | SD | Mean | SD |
| Apse | 2.94E+08 | 1.93E+02 | 4.36E+09 | 3.78E+02 | 6.61E+09 | 1.96E+02 | 2.66E+08 | 1.96E+02 | 1.33E+08 | 1.85E+03 | 5.48E+09 | 9.76E+02 |
| Bulls center | 1.06E+09 | 2.73E+02 | 2.73E+09 | 2.56E+02 | 3.31E+08 | 3.49E+01 | 4.72E+07 | 4.13E+02 | 4.64E+08 | 1.25E+02 | 1.21E+09 | 4.49E+01 |
| Bulls left | 4.50E+09 | 9.48E+01 | 6.12E+09 | 1.20E+03 | 2.83E+09 | 7.93E+02 | 8.18E+07 | 5.33E+01 | 5.16E+09 | 2.16E+02 | 6.64E+09 | 6.12E+02 |
| Bulls wall | 2.79E+09 | 8.10E+02 | 1.99E+08 | 1.40E+02 | 3.53E+09 | 4.48E+02 | 8.65E+06 | 6.15E+01 | 1.95E+09 | 1.85E+01 | 1.19E+08 | 5.01E+02 |
| Airlock-2 wall | 1.93E+09 | 6.53E+01 | 4.20E+09 | 8.05E+01 | 5.51E+09 | 8.67E+01 | 9.64E+07 | 8.66E+02 | 1.59E+09 | 7.47E+01 | 3.57E+09 | 8.63E+02 |
| Nave high | 3.68E+09 | 9.64E+01 | 7.75E+09 | 4.77E+02 | 2.20E+09 | 4.01E+02 | 8.53E+07 | 4.59E+02 | 1.50E+09 | 4.59E+02 | 2.40E+09 | 4.59E+02 |
| Nave low | 5.19E+09 | 1.72E+02 | 2.38E+11 | 3.35E+02 | 1.47E+09 | 2.32E+02 | 5.62E+08 | 8.47E+03 | 1.62E+09 | 7.65E+02 | 1.44E+11 | 8.74E+01 |
| Passage horizontal | 2.46E+09 | 2.12E+02 | 3.40E+09 | 1.36E+01 | 5.35E+08 | 1.84E+01 | 8.73E+07 | 1.09E+02 | 5.44E+08 | 9.20E+01 | 2.86E+09 | 1.02E+02 |
| Passage vertical | 2.56E+10 | 1.19E+02 | 2.44E+09 | 3.36E+01 | 2.64E+10 | 1.28E+02 | 3.30E+07 | 1.28E+03 | 2.63E+09 | 5.11E+03 | 3.44E+09 | 1.29E+01 |

**Table S5.** Pairwise-Adonis comparisons of control and dark zone conditions for bacterial 16S rRNA genes and for fungal ITS region, separately for each sample location. NS, not significant; *$P$ < 0.05; **$P$ < 0.01; ***$P$ < 0.005.

| | **Bacterial 16S rRNA gene** | | | | | **Fungal ITS region** | | | | |
|---|---|---|---|---|---|---|---|---|---|---|
| Sampling location | Df | F Model | $R^2$ | *P* adjusted | | Df | F Model | $R^2$ | *P* adjusted | |
| Apse | 1 | 40.23 | 0.80 | **0.001** | *** | 1 | 9.9 | 0.50 | **0.003** | ** |
| Bulls center | 1 | 15.26 | 0.60 | **0.004** | ** | 1 | 6.49 | 0.39 | **0.003** | ** |
| Bulls left | 1 | 3.16 | 0.24 | **0.011** | * | 1 | 25.14 | 0.72 | **0.003** | ** |
| Bulls wall | 1 | 6.81 | 0.40 | **0.003** | ** | 1 | 3.84 | 0.30 | **0.003** | ** |
| Airlok-2 wall | 1 | 9.72 | 0.49 | **0.004** | ** | 1 | 4.07 | 0.29 | **0.011** | * |
| Nave high | 1 | 55.94 | 0.85 | **0.003** | ** | 1 | 13.02 | 0.57 | **0.003** | ** |
| Nave low | 1 | 16.58 | 0.62 | **0.006** | ** | 1 | 7.94 | 0.47 | **0.003** | ** |
| Passage horizontal | 1 | 13.82 | 0.58 | **0.006** | ** | 1 | 3.65 | 0.27 | **0.005** | ** |
| Passage vertical | 1 | 34.84 | 0.78 | **0.004** | ** | 1 | 2.89 | 0.22 | **0.009** | ** |

**Table S6.** Means and standard deviations (SD) of diversity indices (Simpson 1-D, Shannon H', Evenness index and Chao-1) for bacterial and fungal communities according to the rock surface condition and the sampling area.

| | | Bacteria (16S rRNA gene) | | | | Fungi (ITS2 region) | | | |
|---|---|---|---|---|---|---|---|---|---|
| | | Simpson 1-D | Shannon H' | Evenness | Chao-1 | Simpson 1-D | Shannon H' | Evenness | Chao-1 |
| Apse - Control | Mean | 0.703 | 2.166 | 0.090 | 155.3 | 0.705 | 2.063 | 0.138 | 72.99 |
| | SD | 0.198 | 0.663 | 0.079 | 24.79 | 0.184 | 0.803 | 0.071 | 21.11 |
| Apse - Dark zone | Mean | 0.946 | 3.540 | 0.238 | 162.5 | 0.583 | 1.236 | 0.136 | 30.19 |
| | SD | 0.007 | 0.116 | 0.022 | 18.53 | 0.253 | 0.517 | 0.034 | 9.142 |
| Nave high - Control | Mean | 0.600 | 1.723 | 0.043 | 195.5 | 0.735 | 2.154 | 0.121 | 84.50 |
| | SD | 0.109 | 0.222 | 0.012 | 37.74 | 0.151 | 0.552 | 0.046 | 5.862 |
| Nave high - Dark zone | Mean | 0.932 | 3.242 | 0.165 | 197.5 | 0.508 | 1.092 | 0.088 | 42.55 |
| | SD | 0.023 | 0.407 | 0.055 | 23.70 | 0.195 | 0.407 | 0.025 | 2.808 |
| Nave low - Control | Mean | 0.820 | 2.312 | 0.113 | 120.3 | 0.699 | 1.875 | 0.135 | 61.81 |
| | SD | 0.079 | 0.480 | 0.040 | 18.03 | 0.186 | 0.613 | 0.081 | 5.448 |
| Nave low- Dark zone | Mean | 0.898 | 2.894 | 0.143 | 146.6 | 0.366 | 0.810 | 0.084 | 31.15 |
| | SD | 0.016 | 0.200 | 0.013 | 20.87 | 0.133 | 0.177 | 0.034 | 13.75 |
| Passage horizontal - Control | Mean | 0.875 | 2.788 | 0.108 | 216.6 | 0.542 | 1.424 | 0.074 | 79.14 |
| | SD | 0.060 | 0.663 | 0.058 | 38.64 | 0.237 | 0.578 | 0.031 | 9.973 |
| Passage horizontal - Dark zone | Mean | 0.888 | 3.026 | 0.139 | 188.6 | 0.627 | 1.461 | 0.105 | 50.45 |
| | SD | 0.049 | 0.540 | 0.036 | 28.36 | 0.176 | 0.501 | 0.038 | 12.59 |
| Passage vertical - Control | Mean | 0.849 | 2.575 | 0.090 | 216.1 | 0.648 | 1.866 | 0.110 | 84.02 |
| | SD | 0.033 | 0.358 | 0.035 | 30.07 | 0.232 | 0.993 | 0.072 | 29.39 |
| Passage vertical - Dark zone | Mean | 0.902 | 3.034 | 0.130 | 211.8 | 0.609 | 1.337 | 0.076 | 65.84 |
| | SD | 0.041 | 0.377 | 0.045 | 15.90 | 0.127 | 0.330 | 0.023 | 13.24 |
| Bull center - Control | Mean | 0.817 | 2.729 | 0.069 | 281.4 | 0.785 | 2.104 | 0.132 | 72.18 |
| | SD | 0.067 | 0.385 | 0.020 | 33.47 | 0.052 | 0.330 | 0.018 | 22.40 |
| Bull center - Dark zone | Mean | 0.926 | 3.400 | 0.184 | 209.7 | 0.597 | 1.294 | 0.105 | 48.09 |
| | SD | 0.058 | 0.407 | 0.051 | 28.44 | 0.118 | 0.439 | 0.056 | 11.43 |
| Bull left - Control | Mean | 0.855 | 2.910 | 0.109 | 239.9 | 0.684 | 1.748 | 0.087 | 78.35 |
| | SD | 0.093 | 0.587 | 0.050 | 34.15 | 0.078 | 0.274 | 0.015 | 20.36 |
| Bull left - Dark zone | Mean | 0.939 | 3.470 | 0.180 | 225.0 | 0.530 | 1.113 | 0.072 | 55.64 |
| | SD | 0.020 | 0.256 | 0.045 | 12.28 | 0.156 | 0.447 | 0.031 | 15.98 |
| Bull wall - Control | Mean | 0.868 | 2.98 | 0.112 | 294.5 | 0.647 | 1.941 | 0.126 | 77.45 |
| | SD | 0.080 | 0.775 | 0.082 | 54.32 | 0.305 | 1.089 | 0.079 | 37.13 |
| Bull wall - Dark zone | Mean | 0.870 | 3.029 | 0.147 | 183.9 | 0.729 | 1.905 | 0.122 | 73.60 |
| | SD | 0.094 | 0.651 | 0.052 | 54.20 | 0.123 | 0.494 | 0.046 | 26.22 |
| Airlock-2 wall - Control | Mean | 0.971 | 4.255 | 0.263 | 309.3 | 0.405 | 0.953 | 0.057 | 54.65 |
| | SD | 0.005 | 0.158 | 0.029 | 20.10 | 0.126 | 0.261 | 0.016 | 9.166 |
| Airlock-2 wall - Dark zone | Mean | 0.957 | 3.886 | 0.219 | 254.6 | 0.636 | 1.674 | 0.109 | 64.40 |
| | SD | 0.006 | 0.123 | 0.020 | 11.66 | 0.254 | 0.624 | 0.056 | 10.63 |

**Table S7**. Adonis comparison of rock surface conditions (control vs dark zone) and sample locations across the whole dataset for bacterial 16S rRNA genes and for fungal ITS region.

| | **Bacterial 16S rRNA gene** | | | | **Fungal ITS region** | | | |
|---|---|---|---|---|---|---|---|---|
| | Df | F | *P* | $R^2$ | Df | F | *P* | $R^2$ |
| *Effect :* | | | | | | | | |
| Location | 8 | 13.059 | **0.004** | 0.32 | 8 | 10.792 | **0.001** | 0.36 |
| Rock surface condition | 1 | 53.516 | **0.009** | 0.17 | 1 | 20.818 | **0.009** | 0.08 |
| Location × Rock surface condition | 8 | 5.460 | **0.001** | 0.23 | 8 | 5.460 | **0.001** | 0.18 |
| Residuals | 107 | | | | 105 | | | |

**Table S8.** Pairwise-Adonis comparisons of control and dark zone conditions for bacterial 16S rRNA genes and for fungal ITS region, separately for each.

| | | | **16S Bacterial rRNA gene** | | | | | | **Fungal ITS region** | | | | | |
|---|---|---|---|---|---|---|---|---|---|---|---|---|---|---|
| Rock surface condition | pairs | | Df | Sums Of Sqs | F | $R^2$ | *P* | *P* adjusted | Df | Sums Of Sqs | F | $R^2$ | *P* | *P* adjusted |
| CONTROL | Apse | Bulls_center | 1 | 0.78 | 10.96 | 0.52 | 0.004 | 0.144 | 1 | 1.76 | 13.05 | 0.57 | 0.004 | 0.144 |
| | Apse | Bulls_left | 1 | 2.01 | 14.95 | 0.60 | 0.002 | 0.072 | 1 | 1.55 | 12.89 | 0.56 | 0.005 | 0.180 |
| | Apse | Airlock-2_Bulls | 1 | 1.24 | 10.52 | 0.51 | 0.001 | **0.036** | 1 | 1.37 | 10.30 | 0.53 | 0.001 | **0.036** |
| | Apse | Airlock-2 | 1 | 2.27 | 25.61 | 0.72 | 0.003 | 0.108 | 1 | 1.44 | 8.60 | 0.46 | 0.002 | 0.072 |
| | Apse | Nave_high | 1 | 0.11 | 2.68 | 0.21 | 0.046 | 1.000 | 1 | 0.62 | 3.52 | 0.26 | 0.006 | 0.216 |
| | Apse | Nave_low | 1 | 0.78 | 8.78 | 0.47 | 0.001 | **0.036** | 1 | 0.75 | 3.55 | 0.26 | 0.013 | 0.468 |
| | Apse | Passage_horizontal | 1 | 1.35 | 24.38 | 0.71 | 0.007 | 0.252 | 1 | 1.18 | 7.11 | 0.42 | 0.003 | 0.108 |
| | Apse | Passage_vertical | 1 | 1.51 | 23.10 | 0.70 | 0.004 | 0.144 | 1 | 1.20 | 7.99 | 0.44 | 0.003 | 0.108 |
| | Bulls_center | Bulls_left | 1 | 1.63 | 11.04 | 0.52 | 0.002 | 0.072 | 1 | 1.74 | 22.40 | 0.69 | 0.003 | 0.108 |
| | Bulls_center | Airlock-2_Bulls | 1 | 1.06 | 8.06 | 0.45 | 0.003 | 0.108 | 1 | 0.55 | 6.50 | 0.42 | 0.004 | 0.144 |
| | Bulls_center | Airlock-2 | 1 | 2.01 | 19.74 | 0.66 | 0.002 | 0.072 | 1 | 2.06 | 16.50 | 0.62 | 0.006 | 0.216 |
| | Bulls_center | Nave_high | 1 | 0.71 | 12.71 | 0.56 | 0.005 | 0.180 | 1 | 1.60 | 11.92 | 0.54 | 0.002 | 0.072 |
| | Bulls_center | Nave_low | 1 | 0.88 | 8.56 | 0.46 | 0.003 | 0.108 | 1 | 1.52 | 9.06 | 0.48 | 0.003 | 0.108 |
| | Bulls_center | Passage_horizontal | 1 | 0.74 | 10.83 | 0.52 | 0.002 | 0.072 | 1 | 0.75 | 6.12 | 0.38 | 0.002 | 0.072 |
| | Bulls_center | Passage_vertical | 1 | 1.25 | 15.85 | 0.61 | 0.004 | 0.144 | 1 | 0.62 | 5.85 | 0.37 | 0.002 | 0.072 |
| | Bulls_left | Airlock-2_Bulls | 1 | 1.34 | 6.86 | 0.41 | 0.004 | 0.144 | 1 | 1.50 | 21.52 | 0.71 | 0.002 | 0.072 |
| | Bulls_left | Airlock-2 | 1 | 1.13 | 6.81 | 0.41 | 0.002 | 0.072 | 1 | 0.99 | 8.94 | 0.47 | 0.003 | 0.108 |
| | Bulls_left | Nave_high | 1 | 2.14 | 17.97 | 0.64 | 0.002 | 0.072 | 1 | 1.91 | 15.97 | 0.61 | 0.004 | 0.144 |
| | Bulls_left | Nave_low | 1 | 1.89 | 11.44 | 0.53 | 0.005 | 0.180 | 1 | 1.80 | 11.71 | 0.54 | 0.004 | 0.144 |
| | Bulls_left | Passage_horizontal | 1 | 1.49 | 11.32 | 0.53 | 0.002 | 0.072 | 1 | 1.73 | 15.89 | 0.61 | 0.003 | 0.108 |
| | Bulls_left | Passage_vertical | 1 | 1.39 | 9.77 | 0.49 | 0.005 | 0.180 | 1 | 1.53 | 16.49 | 0.62 | 0.004 | 0.144 |
| | Airlock-2_Bulls | Airlock-2 | 1 | 1.04 | 6.99 | 0.41 | 0.002 | 0.072 | 1 | 1.59 | 13.00 | 0.59 | 0.003 | 0.108 |
| | Airlock-2_Bulls | Nave_high | 1 | 1.26 | 12.27 | 0.55 | 0.003 | 0.108 | 1 | 1.35 | 10.23 | 0.53 | 0.003 | 0.108 |
| | Airlock-2_Bulls | Nave_low | 1 | 1.18 | 7.90 | 0.44 | 0.002 | 0.072 | 1 | 1.19 | 7.02 | 0.44 | 0.002 | 0.072 |
| | Airlock-2_Bulls | Passage_horizontal | 1 | 1.27 | 10.95 | 0.52 | 0.002 | 0.072 | 1 | 1.45 | 12.07 | 0.57 | 0.008 | 0.288 |
| | Airlock-2_Bulls | Passage_vertical | 1 | 1.33 | 10.53 | 0.51 | 0.005 | 0.180 | 1 | 1.11 | 10.88 | 0.55 | 0.002 | 0.072 |
| | Airlock-2 | Nave_high | 1 | 2.48 | 33.73 | 0.77 | 0.003 | 0.108 | 1 | 1.92 | 11.47 | 0.53 | 0.004 | 0.144 |
| | Airlock-2 | Nave_low | 1 | 2.26 | 18.83 | 0.65 | 0.005 | 0.180 | 1 | 1.76 | 8.76 | 0.47 | 0.004 | 0.144 |
| | Airlock-2 | Passage_horizontal | 1 | 2.16 | 25.01 | 0.71 | 0.003 | 0.108 | 1 | 2.01 | 12.84 | 0.56 | 0.003 | 0.108 |
| | Airlock-2 | Passage_vertical | 1 | 2.13 | 22.01 | 0.69 | 0.004 | 0.144 | 1 | 1.90 | 13.57 | 0.58 | 0.004 | 0.144 |
| | Nave_high | Nave_low | 1 | 0.58 | 7.84 | 0.44 | 0.004 | 0.144 | 1 | 0.52 | 2.49 | 0.20 | 0.040 | 1.000 |
| | Nave_high | Passage_horizontal | 1 | 1.12 | 27.77 | 0.74 | 0.005 | 0.180 | 1 | 1.10 | 6.63 | 0.40 | 0.005 | 0.180 |
| | Nave_high | Passage_vertical | 1 | 1.40 | 27.68 | 0.73 | 0.004 | 0.144 | 1 | 1.12 | 7.51 | 0.43 | 0.003 | 0.108 |
| | Nave_low | Passage_horizontal | 1 | 0.54 | 6.29 | 0.39 | 0.001 | **0.036** | 1 | 1.17 | 5.89 | 0.37 | 0.005 | 0.180 |
| | Nave_low | Passage_vertical | 1 | 0.81 | 8.31 | 0.45 | 0.003 | 0.108 | 1 | 1.20 | 6.58 | 0.40 | 0.004 | 0.144 |
| | Passage_horizontal | Passage_vertical | 1 | 0.24 | 3.85 | 0.28 | 0.010 | 0.360 | 1 | 0.10 | 0.75 | 0.07 | 0.582 | 1.000 |
| DARK ZONE | Apse | Bulls_center | 1 | 1.35 | 12.06 | 0.55 | 0.006 | 0.216 | 1 | 1.92 | 19.70 | 0.66 | 0.004 | 0.144 |
| | Apse | Bulls_left | 1 | 1.41 | 9.50 | 0.49 | 0.002 | 0.072 | 1 | 1.81 | 17.60 | 0.64 | 0.003 | 0.108 |
| | Apse | Airlock-2_Bulls | 1 | 1.87 | 12.93 | 0.56 | 0.002 | 0.072 | 1 | 1.31 | 6.27 | 0.41 | 0.002 | 0.072 |
| | Apse | Airlock-2 | 1 | 2.01 | 26.64 | 0.73 | 0.003 | 0.108 | 1 | 1.33 | 7.22 | 0.42 | 0.003 | 0.108 |
| | Apse | Nave_high | 1 | 1.84 | 38.73 | 0.79 | 0.007 | 0.252 | 1 | 1.53 | 9.90 | 0.50 | 0.002 | 0.072 |
| | Apse | Nave_low | 1 | 1.80 | 20.37 | 0.67 | 0.002 | 0.072 | 1 | 1.50 | 12.97 | 0.56 | 0.005 | 0.180 |
| | Apse | Passage_horizontal | 1 | 1.42 | 12.64 | 0.56 | 0.004 | 0.144 | 1 | 1.28 | 6.23 | 0.38 | 0.005 | 0.180 |
| | Apse | Passage_vertical | 1 | 1.99 | 39.76 | 0.80 | 0.003 | 0.108 | 1 | 1.52 | 15.30 | 0.60 | 0.002 | 0.072 |
| | Bulls_center | Bulls_left | 1 | 0.42 | 2.09 | 0.17 | 0.025 | 0.900 | 1 | 0.05 | 0.68 | 0.06 | 0.496 | 1.000 |
| | Bulls_center | Airlock-2_Bulls | 1 | 0.88 | 4.44 | 0.31 | 0.001 | **0.036** | 1 | 1.01 | 5.86 | 0.39 | 0.001 | **0.036** |
| | Bulls_center | Airlock-2 | 1 | 1.31 | 10.09 | 0.50 | 0.005 | 0.180 | 1 | 1.67 | 10.94 | 0.52 | 0.004 | 0.144 |
| | Bulls_center | Nave_high | 1 | 1.30 | 12.78 | 0.56 | 0.005 | 0.180 | 1 | 0.56 | 4.58 | 0.31 | 0.009 | 0.324 |
| | Bulls_center | Nave_low | 1 | 0.62 | 4.35 | 0.30 | 0.001 | **0.036** | 1 | 0.54 | 6.53 | 0.40 | 0.004 | 0.144 |
| | Bulls_center | Passage_horizontal | 1 | 0.99 | 5.90 | 0.37 | 0.001 | **0.036** | 1 | 0.75 | 4.32 | 0.30 | 0.005 | 0.180 |
| | Bulls_center | Passage_vertical | 1 | 0.91 | 8.74 | 0.47 | 0.004 | 0.144 | 1 | 0.39 | 5.81 | 0.37 | 0.004 | 0.144 |
| | Bulls_left | Airlock-2_Bulls | 1 | 0.84 | 3.57 | 0.26 | 0.010 | 0.360 | 1 | 1.09 | 6.08 | 0.40 | 0.005 | 0.180 |
| | Bulls_left | Airlock-2 | 1 | 0.91 | 5.47 | 0.35 | 0.003 | 0.108 | 1 | 1.65 | 10.41 | 0.51 | 0.002 | 0.072 |
| | Bulls_left | Nave_high | 1 | 1.41 | 10.23 | 0.51 | 0.004 | 0.144 | 1 | 0.50 | 3.94 | 0.28 | 0.012 | 0.432 |
| | Bulls_left | Nave_low | 1 | 0.78 | 4.35 | 0.30 | 0.005 | 0.180 | 1 | 0.43 | 4.81 | 0.32 | 0.015 | 0.540 |
| | Bulls_left | Passage_horizontal | 1 | 0.63 | 3.11 | 0.24 | 0.027 | 0.972 | 1 | 0.76 | 4.28 | 0.30 | 0.004 | 0.144 |
| | Bulls_left | Passage_vertical | 1 | 0.50 | 3.55 | 0.26 | 0.003 | 0.108 | 1 | 0.37 | 5.09 | 0.34 | 0.006 | 0.216 |
| | Airlock-2_Bulls | Airlock-2 | 1 | 0.74 | 4.54 | 0.31 | 0.015 | 0.540 | 1 | 0.77 | 2.85 | 0.24 | 0.005 | 0.180 |
| | Airlock-2_Bulls | Nave_high | 1 | 1.66 | 12.34 | 0.55 | 0.004 | 0.144 | 1 | 1.02 | 4.30 | 0.32 | 0.012 | 0.432 |
| | Airlock-2_Bulls | Nave_low | 1 | 0.82 | 4.68 | 0.32 | 0.003 | 0.108 | 1 | 1.19 | 6.17 | 0.41 | 0.002 | 0.072 |
| | Airlock-2_Bulls | Passage_horizontal | 1 | 1.36 | 6.80 | 0.40 | 0.003 | 0.108 | 1 | 0.85 | 2.91 | 0.24 | 0.003 | 0.108 |
| | Airlock-2_Bulls | Passage_vertical | 1 | 1.27 | 9.21 | 0.48 | 0.005 | 0.180 | 1 | 1.05 | 6.00 | 0.40 | 0.003 | 0.108 |
| | Airlock-2 | Nave_high | 1 | 2.01 | 30.92 | 0.76 | 0.003 | 0.108 | 1 | 1.11 | 5.30 | 0.35 | 0.016 | 0.576 |
| | Airlock-2 | Nave_low | 1 | 1.40 | 13.18 | 0.57 | 0.003 | 0.108 | 1 | 1.58 | 9.26 | 0.48 | 0.006 | 0.216 |
| | Airlock-2 | Passage_horizontal | 1 | 1.36 | 10.44 | 0.51 | 0.004 | 0.144 | 1 | 0.94 | 3.59 | 0.26 | 0.007 | 0.252 |
| | Airlock-2 | Passage_vertical | 1 | 1.33 | 19.70 | 0.66 | 0.001 | **0.036** | 1 | 1.29 | 8.29 | 0.45 | 0.007 | 0.252 |
| | Nave_high | Nave_low | 1 | 1.21 | 15.43 | 0.61 | 0.003 | 0.108 | 1 | 0.21 | 1.53 | 0.13 | 0.099 | 1.000 |
| | Nave_high | Passage_horizontal | 1 | 1.79 | 17.46 | 0.64 | 0.003 | 0.108 | 1 | 1.07 | 4.63 | 0.32 | 0.005 | 0.180 |
| | Nave_high | Passage_vertical | 1 | 2.05 | 51.32 | 0.84 | 0.001 | **0.036** | 1 | 1.20 | 9.58 | 0.49 | 0.003 | 0.108 |
| | Nave_low | Passage_horizontal | 1 | 1.24 | 8.66 | 0.46 | 0.005 | 0.180 | 1 | 1.23 | 6.41 | 0.39 | 0.003 | 0.108 |
| | Nave_low | Passage_vertical | 1 | 1.11 | 13.74 | 0.58 | 0.001 | **0.036** | 1 | 1.35 | 15.79 | 0.61 | 0.007 | 0.252 |
| | Passage_horizontal | Passage_vertical | 1 | 0.64 | 6.12 | 0.38 | 0.003 | 0.108 | 1 | 0.56 | 3.21 | 0.24 | 0.006 | 0.216 |

**Table S9.** Computation of the 47 similarity scores for dark zones of Lascaux, in relation to the situation of the Apse's dark zones.

**Apse**

| | Raw data: Unmarked surface | Raw data: Unmarked surfaces (SD) | Raw data: Dark zone | Raw data: Dark zone (SD) | Log data: Unmarked surface (log) | Log data: Dark zone (log) | Log data: Delta log | Score |
|---|---|---|---|---|---|---|---|---|
| **Bacteria** | | | | | | | | |
| *Niabella* | 1 | 0.0 | 778 | 13.1 | 0.000 | 2.890 | 2.890 | 1 |
| *Steroidobacter* | 8 | 3.2 | 2067 | 172.9 | 0.903 | 3.315 | 2.412 | 1 |
| *Acidothermus* | 4 | 1.0 | 826 | 103.2 | 0.602 | 2.916 | 2.314 | 1 |
| *Phaselicystis* | 3 | 0.0 | 506 | 58.4 | 0.477 | 2.704 | 2.227 | 1 |
| *Saccharopolyspora* | 8 | 1.8 | 975 | 168.8 | 0.903 | 2.989 | 2.085 | 1 |
| *Nitrospira* | 21 | 7.6 | 2467 | 220.4 | 1.322 | 3.392 | 2.069 | 1 |
| *Jatrophihabitans* | 2 | 0.0 | 110 | 5.6 | 0.301 | 2.041 | 1.740 | 1 |
| *Nonomuraea* | 245 | 89.2 | 11017 | 354.9 | 2.389 | 4.042 | 1.652 | 1 |
| *Tundrisphaera* | 23 | 5.7 | 1023 | 142.3 | 1.361 | 3.009 | 1.648 | 1 |
| *Bryobacter* | 154 | 54.1 | 6629 | 214.8 | 2.187 | 3.821 | 1.633 | 1 |
| *Flavihumibacter* | 17 | 6.9 | 658 | 94.2 | 1.230 | 2.818 | 1.587 | 1 |
| *Actinophytocola* | 19 | 6.8 | 651 | 42.0 | 1.278 | 2.813 | 1.534 | 1 |
| *Alysiosphaera* | 4 | 0.0 | 126 | 0.0 | 0.602 | 2.100 | 1.498 | 1 |
| *Galbitalea* | 1 | 0.4 | 31 | 4.2 | 0.000 | 1.491 | 1.491 | 1 |
| *Microlunatus* | 2 | 0.4 | 55 | 5.4 | 0.301 | 1.740 | 1.439 | 1 |
| Unidentified (Solirubrobactera | 29 | 0.0 | 637 | 5.4 | 1.462 | 2.804 | 1.341 | 1 |
| Unidentified (Thermomicrobia | 21 | 0.0 | 323 | 0.0 | 1.322 | 2.509 | 1.186 | 1 |
| *Jiangella* | 18 | 2.5 | 230 | 48.5 | 1.255 | 2.361 | 1.106 | 1 |
| *Sphingopyxis* | 323 | 2.5 | 3663 | 48.5 | 2.509 | 3.563 | 1.054 | 1 |
| *Afipia* | 72 | 10.8 | 771 | 18.0 | 1.857 | 2.887 | 1.029 | 1 |
| *Phenylobacterium* | 25 | 2.7 | 255 | 41.0 | 1.397 | 2.406 | 1.008 | 1 |
| *Methylopila* | 205 | 61.4 | 15 | 3.0 | 2.311 | 1.176 | -1.135 | 1 |
| *Streptomyces* | 1163 | 161.5 | 66 | 1.8 | 3.065 | 1.819 | -1.246 | 1 |
| *Reyranella* | 368 | 28.5 | 19 | 6.7 | 2.565 | 1.278 | -1.287 | 1 |
| *Rhodopseudomonas* | 195 | 49.1 | 3 | 0.8 | 2.290 | 0.477 | -1.812 | 1 |
| Unidentified (Alcaligenaceae) | 2721 | 6.8 | 17 | 2.0 | 3.434 | 1.230 | -2.204 | 1 |
| *Pseudomonas* | 50870 | 3896.6 | 41 | 4.7 | 4.706 | 1.612 | -3.093 | 1 |
| Bray-curtis distance | NA | NA | NA | NA | NA | NA | NA | 1 |
| 16S Copy number / ng DNA | 2.94E+08 | 193.2 | 4.36E+09 | 378.1 | 8.468 | 9.639 | 1.171 | 1 |
| **Archaea** | | | | | | | | |
| Number of reads | 8129 | 431.2 | 745 | 5.2 | 3.910 | 2.872 | -1.037 | 1 |
| 16S Copy number / ng DNA | 6.61E+9 | 195.8 | 2.66E+08 | 195.8 | 9.820 | 6.958 | -2.861 | 1 |
| **Fungi** | | | | | | | | |
| *Chrysosporium* | 2666 | 542.5 | 1 | 0.0 | 3.425 | 0.000 | -3.425 | 1 |
| *Pseudogymnoascus* | 17486 | 1910.9 | 18 | 0.8 | 4.242 | 1.255 | -2.987 | 1 |
| *Illyonectria* | 882 | 190.1 | 2 | 0.0 | 2.945 | 0.301 | -2.644 | 1 |
| *Trichoderma* | 597 | 77.7 | 2 | 0.8 | 2.775 | 0.301 | -2.474 | 1 |
| *Serendipita* | 1046 | 338.0 | 9 | 0.0 | 3.019 | 0.954 | -2.065 | 1 |
| *Cephalotrichum* | 1068 | 114.4 | 12 | 1.8 | 3.028 | 1.079 | -1.949 | 1 |
| Unidentified (Hypocreales) | 1086 | 178.0 | 26 | 7.1 | 3.035 | 1.414 | -1.620 | 1 |
| *Oidodendron* | 296 | 39.0 | 14 | 3.0 | 2.471 | 1.146 | -1.325 | 1 |
| Unidentified | 9764 | 3235.6 | 637 | 74.8 | 3.989 | 2.804 | -1.185 | 1 |
| *Clonostachys* | 719 | 107.5 | 64 | 15.4 | 2.856 | 1.806 | -1.050 | 1 |
| *Gliomastix* | 895 | 63.2 | 87 | 13.6 | 2.951 | 1.939 | -1.012 | 1 |
| *Ochroconis* | 3003 | 57.5 | 38910 | 765.0 | 3.477 | 4.590 | 1.112 | 1 |
| *Mortierella* | 55 | 10.5 | 9100 | 1408.1 | 1.740 | 3.959 | 2.218 | 1 |
| *Saccharomyces* | 1 | 0.0 | 212 | 1.7 | 0.000 | 2.326 | 2.326 | 1 |
| Bray-curtis distance | NA | NA | NA | NA | NA | NA | NA | 1 |
| ITS Copy number / ng DNA | 1.33E+08 | 1851.8 | 5.48E+09 | 975.8 | 8.123 | 9.738 | 1.615 | 1 |

**Nave high**

| | Raw data: Unmarked surface | Raw data: Unmarked surfaces (SD) | Raw data: Dark zone | Raw data: Dark zone (SD) | Log data: Unmarked surface (log) | Log data: Dark zone (log) | Log data: Delta log | Score |
|---|---|---|---|---|---|---|---|---|
| **Bacteria** | | | | | | | | |
| *Niabella* | 1 | 0.0 | 1 | 0.0 | 0.000 | 0.000 | 0.000 | 0.0 |
| *Steroidobacter* | 0 | 0.0 | 0 | 0.0 | NA | NA | NA | 0.0 |
| *Acidothermus* | 0 | 0.0 | 0 | 0.0 | NA | NA | NA | 0.0 |
| *Phaselicystis* | 2 | 0.5 | 2 | 0.8 | 0.301 | 0.301 | 0,000 | 0.0 |
| *Saccharopolyspora* | 5 | 0.0 | 14 | 0.0 | 0.698 | 1.146 | 0.447 | 0.4 |
| *Nitrospira* | 2 | 0.0 | 400 | 115.7 | 0.301 | 2.602 | 2.301 | 0.9 |
| *Jatrophihabitans* | 13 | 1.8 | 4 | 1.2 | 1.113 | 0.602 | -0.511 | |
| *Nonomuraea* | 16 | 1.6 | 448 | 110.1 | 1.204 | 2.651 | 1.447 | 1.0 |
| *Tundrisphaera* | 2 | 0.0 | 50 | 0.0 | 0.301 | 1.698 | 1.397 | 0.9 |
| *Bryobacter* | 9 | 1.2 | 29 | 7.0 | 0.954 | 1.462 | 0.508 | 0.5 |
| *Flavihumibacter* | 0 | 0.0 | 1 | 0.0 | NA | 0.000 | NA | 0.0 |
| *Actinophytocola* | 0 | 0.0 | 7 | 1.1 | NA | 0.845 | NA | 0.0 |
| *Alysiosphaera* | 0 | 0.0 | 5 | 0.0 | NA | 0.698 | NA | 0.0 |
| *Galbitalea* | 0 | 0.0 | 2 | 0.8 | NA | 0.301 | NA | 0.0 |
| *Microlunatus* | 1 | 0.0 | 2 | 0.8 | 0.000 | 0.301 | 0.301 | 0.3 |
| Unidentified (Solirubrobactera | 110 | 0.8 | 21 | 0.5 | 2.041 | 1.322 | -0.719 | |
| Unidentified (Thermomicrobia | 164 | 0.0 | 194 | 0.0 | 2.214 | 2.287 | 0.0729 | 0.0 |
| *Jiangella* | 2 | 0.5 | 482 | 174.5 | 0.301 | 2.683 | 2.382 | 0.0 |
| *Sphingopyxis* | 15 | 0.5 | 4054 | 84.5 | 1.176 | 3.607 | 2.431 | 0.0 |
| *Afipia* | 57 | 5.8 | 1115 | 95.2 | 1.755 | 3.047 | 1.291 | 0.9 |
| *Phenylobacterium* | 9 | 1.2 | 22 | 8.5 | 0.954 | 1.342 | 0.388 | 0.4 |
| *Methylopila* | 75 | 20.4 | 961 | 62.2 | 1.875 | 2.982 | 1.107 | |
| *Streptomyces* | 394 | 20.6 | 77 | 7.2 | 2.595 | 1.886 | -0.709 | 0.7 |
| *Reyranella* | 7 | 1.6 | 0 | 0.0 | 0.845 | NA | NA | 0.0 |
| *Rhodopseudomonas* | 0 | 0.0 | 0 | 0.0 | NA | NA | NA | 0.0 |
| Unidentified (Alcaligenaceae) | 8578 | 0.0 | 7891 | 1.1 | 3.933 | 3.897 | -0.036 | 0.0 |
| *Pseudomonas* | 63810 | 1056.8 | 9753 | 758.3 | 4.804 | 3.989 | -0.815 | 0.3 |
| Bray-curtis distance | 0.36 | NA | 0.77 | NA | NA | NA | 0.403 | 0.0 |
| 16S Copy number / ng DNA | 3.68E+09 | 96.4 | 7.75E+09 | 477.0 | 0.322 | 9.889 | 0.411 | 0.4 |
| **Archaea** | | | | | | | | |
| Number of reads | 17770 | 732.8 | 1798 | 16.1 | 4.249 | 3.254 | -0.994 | 1.0 |
| 16S Copy number / ng DNA | 2.20E+09 | 400.7 | 8.53E+07 | 458.9 | 9.341 | 6.209 | -2.666 | 0.0 |
| **Fungi** | | | | | | | | |
| *Chrysosporium* | 2413 | 378.1 | 8 | 5.4 | 3.382 | 0.903 | -2.479 | 0.8 |
| *Pseudogymnoascus* | 13827 | 684.5 | 90 | 19.0 | 4.140 | 1.954 | -2.186 | 0.8 |
| *Illyonectria* | 10 | 2.8 | 0 | 0.0 | 1.000 | NA | NA | 0.0 |
| *Trichoderma* | 309 | 32.3 | 2827 | 18.1 | 2.489 | 3.451 | 0.961 | |
| *Serendipita* | 8 | 0.0 | 0 | 0.0 | 0.903 | NA | NA | 0.0 |
| *Cephalotrichum* | 1651 | 209.1 | 27 | 9.8 | 3.217 | 1.431 | -1.786 | 0.9 |
| Unidentified (Hypocreales) | 4337 | 632.1 | 4567 | 235.6 | 3.637 | 3.659 | 0.022 | |
| *Oidodendron* | 25 | 2.5 | 1 | 0.0 | 1.397 | 0.000 | -1.397 | 1.0 |
| Unidentified | 3178 | 668.6 | 184 | 8.8 | 3.502 | 2.264 | -1.237 | 1.0 |
| *Clonostachys* | 200 | 38.7 | 6 | 0.6 | 2.301 | 0.778 | -1.522 | 0.6 |
| *Gliomastix* | 355 | 58.8 | 13 | 8.6 | 2.550 | 1.113 | -1.436 | 0.6 |
| *Ochroconis* | 913 | 210.9 | 627 | 213.3 | 2.960 | 2.797 | -0.163 | |
| *Mortierella* | 774 | 90.1 | 67 | 2.3 | 2.888 | 1.826 | -1.062 | |
| *Saccharomyces* | 173 | 0.0 | 1 | 0.0 | 2.238 | 0.000 | -2.238 | |
| Bray-curtis distance | 0.54 | NA | 0.847 | NA | NA | NA | 0.568 | 0.5 |
| ITS Copy number / ng DNA | 1.50E+09 | 458.9 | 2.40E+09 | 458.9 | 9.176 | 9.380 | 0.416 | 0.0 |

**Nave low**

| | Raw data: Unmarked surface | Raw data: Unmarked surfaces (SD) | Raw data: Dark zone | Raw data: Dark zone (SD) | Log data: Unmarked surface (log) | Log data: Dark zone (log) | Log data: Delta log | Score |
|---|---|---|---|---|---|---|---|---|
| **Bacteria** | | | | | | | | |
| *Niabella* | 0 | 0.0 | 0 | 0.0 | NA | NA | NA | 0.0 |
| *Steroidobacter* | 1 | 0.0 | 0 | 0.0 | NA | NA | NA | 0.0 |
| *Acidothermus* | 3 | 0.8 | 0 | 0.0 | 0.477 | NA | NA | 0.0 |
| *Phaselicystis* | 36 | 14.6 | 55 | 21.9 | 1.556 | 1.740 | 0.184 | 0.0 |
| *Saccharopolyspora* | 25 | 10.2 | 3 | 0.0 | 1.397 | 0.477 | -0.920 | |
| *Nitrospira* | 104 | 11.8 | 21 | 0.0 | 2.017 | 1.322 | -0.694 | |
| *Jatrophihabitans* | 3 | 1.2 | 0 | 0.0 | 0.477 | NA | NA | 0.0 |
| *Nonomuraea* | 772 | 312.1 | 50 | 8.6 | 2.887 | 1.690 | -1.188 | |
| *Tundrisphaera* | 72 | 2.8 | 3 | 0.4 | 1.857 | 0.477 | -1.380 | |
| *Bryobacter* | 448 | 182.8 | 73 | 22.0 | 2.651 | 1.863 | -0.787 | |
| *Flavihumibacter* | 164 | 66.9 | 170 | 69.4 | 2.214 | 2.230 | 0.015 | 0.0 |
| *Actinophytocola* | 36 | 14.2 | 154 | 41.1 | 1.556 | 2.187 | 0.631 | 0.5 |
| *Alysiosphaera* | 1 | 0.0 | 0 | 0.0 | NA | NA | NA | 0.0 |
| *Galbitalea* | 0 | 0.0 | 0 | 0.0 | NA | NA | NA | 0.0 |
| *Microlunatus* | 8 | 3.2 | 0 | 0.0 | NA | NA | NA | 0.0 |
| Unidentified (Solirubrobactera | 8 | 0.0 | 1 | 0.0 | 0.903 | 0.000 | -0.903 | |
| Unidentified (Thermomicrobia | 69 | 0.0 | 34 | 0.0 | 1.838 | 1.531 | -0.307 | |
| *Jiangella* | 161 | 65.7 | 18 | 4.3 | 2.206 | 1.255 | -0.951 | |
| *Sphingopyxis* | 722 | 65.8 | 18024 | 45.0 | 2.858 | 4.255 | 1.397 | 0.8 |
| *Afipia* | 407 | 159.8 | 9101 | 630.0 | 2.609 | 3.959 | 1.349 | 0.8 |
| *Phenylobacterium* | 29 | 10.8 | 135 | 40.0 | 1.462 | 2.130 | 0.667 | 0.7 |
| *Methylopila* | 163 | 66.5 | 431 | 38.7 | 2.212 | 2.634 | 0.422 | |
| *Streptomyces* | 56 | 8.9 | 142 | 26.8 | 1.748 | 2.152 | 0.404 | |
| *Reyranella* | 12 | 3.5 | 4 | 1.6 | 1.079 | 0.602 | -0.477 | 0.4 |
| *Rhodopseudomonas* | 0 | 0.0 | 0 | 0.0 | NA | NA | NA | 0.0 |
| Unidentified (Alcaligenaceae) | 16947 | 14.2 | 1447 | 41.1 | 4.229 | 3.160 | -1.068 | 0.5 |
| *Pseudomonas* | 34436 | 4598.8 | 235 | 21.3 | 4.537 | 2.371 | -2.165 | 0.8 |
| Bray-curtis distance | 0.523 | NA | 0.829 | NA | NA | NA | 21.500 | 0.0 |
| 16S Copy number / ng DNA | 5.19E+09 | 171.5 | 2.33E+11 | 335.3 | 1.661 | 11.377 | 0.133 | 0.0 |
| **Archaea** | | | | | | | | |
| Number of reads | 14482 | 1919.7 | 433 | 25.9 | 4.160 | 2.636 | -1.524 | 0.7 |
| 16S Copy number / ng DNA | 1.47E+09 | 232.4 | 5.62E+08 | 8471.4 | 9.166 | 7.497 | -3.266 | 0.5 |
| **Fungi** | | | | | | | | |
| *Chrysosporium* | 13 | 3.0 | 2.4 | 0.0 | 1.113 | 0.380 | -0.733 | 0.3 |
| *Pseudogymnoascus* | 19252 | 4113.5 | 4.8 | 3.5 | 4.284 | 0.681 | -3.603 | 1.0 |
| *Illyonectria* | 138 | 42.0 | 0 | 0.0 | 2.139 | NA | NA | 0.0 |
| *Trichoderma* | 6245 | 467.0 | 474 | 26.6 | 3.795 | 2.675 | -1.119 | 0.5 |
| *Serendipita* | 0 | 0.0 | 0 | 0.0 | NA | NA | NA | 0.0 |
| *Cephalotrichum* | 5856 | 63.2 | 9775.2 | 68.5 | 3.767 | 3.990 | 0.222 | |
| Unidentified (Hypocreales) | 1658 | 126.0 | 188.4 | 66.4 | 3.219 | 2.275 | -0.944 | 0.6 |
| *Oidodendron* | 20 | 0.0 | 0 | 0.0 | 1.301 | NA | NA | 0.0 |
| Unidentified | 1425 | 436.1 | 93.6 | 3.9 | 3.153 | 1.971 | -1.182 | 1.0 |
| *Clonostachys* | 326 | 123.8 | 2.4 | 0.0 | 2.513 | 0.380 | -2.133 | 0.0 |
| *Gliomastix* | 548 | 81.8 | 3.6 | 1.4 | 2.738 | 0.556 | -2.182 | 0.0 |
| *Ochroconis* | 121 | 58.0 | 6066 | 3.8 | 2.082 | 3.782 | 1.700 | 0.6 |
| *Mortierella* | 1921 | 154.6 | 32.4 | 3.1 | 3.283 | 1.510 | -1.772 | |
| *Saccharomyces* | 985 | 318.0 | 0 | 0.0 | 2.993 | NA | NA | 0.0 |
| Bray-curtis distance | 0.546 | NA | 0.806 | NA | NA | NA | 0.485 | 0.6 |
| ITS Copy number / ng DNA | 1.62E+09 | 765.4 | 1.44E+11 | 87.4 | 9.209 | 11.156 | 0.1099 | 0.8 |

**Passage horizontal**

| | Raw data: Unmarked surface | Raw data: Unmarked surfaces (SD) | Raw data: Dark zone | Raw data: Dark zone (SD) | Log data: Unmarked surface (log) | Log data: Dark zone (log) | Log data: Delta log | Score |
|---|---|---|---|---|---|---|---|---|
| **Bacteria** | | | | | | | | |
| *Niabella* | 0 | 0.0 | 0 | 0.0 | NA | NA | NA | 0.0 |
| *Steroidobacter* | 1 | 0.0 | 0 | 0.0 | 0.000 | NA | NA | 0.0 |
| *Acidothermus* | 0 | 0.0 | 0 | 0.0 | NA | NA | NA | 0.0 |
| *Phaselicystis* | 2 | 0.8 | 102 | 29.5 | 0.301 | 2.008 | 1.707 | 0.8 |
| *Saccharopolyspora* | 30 | 0.0 | 24 | 0.0 | 1.477 | 1.380 | -0.096 | |
| *Nitrospira* | 33 | 1.6 | 838 | 161.2 | 1.518 | 2.923 | 1.404 | 0.9 |
| *Jatrophihabitans* | 27 | 6.8 | 351 | 75.9 | 1.431 | 2.545 | 1.113 | 0.8 |
| *Nonomuraea* | 6 | 1.1 | 19 | 2.8 | 0.778 | 1.278 | 0.500 | 0.5 |
| *Tundrisphaera* | 2 | 0.0 | 10 | 0.0 | 0.301 | 1.000 | 0.698 | 0.6 |
| *Bryobacter* | 41 | 10.9 | 1583 | 202.5 | 1.612 | 3.199 | 1.586 | 1.0 |
| *Flavihumibacter* | 0 | 0.0 | 0 | 0.0 | NA | NA | NA | 0.0 |
| *Actinophytocola* | 5 | 0.7 | 46 | 6.5 | 0.698 | 1.662 | 0.963 | 0.7 |
| *Alysiosphaera* | 2 | 0.0 | 0 | 1.1 | 0.301 | NA | NA | 0.0 |
| *Galbitalea* | 0 | 0.0 | 0 | 0.0 | NA | NA | NA | 0.0 |
| *Microlunatus* | 18 | 3.1 | 397 | 74.0 | 1.255 | 2.598 | 1.343 | 1.0 |
| Unidentified (Solirubrobactera | 15 | 0.9 | 173 | 7.8 | 1.176 | 2.238 | 1.061 | 0.8 |
| Unidentified (Thermomicrobia | 24 | 0.0 | 329 | 0.0 | 1.380 | 2.517 | 1.136 | 1.0 |
| *Jiangella* | 6 | 1.5 | 715 | 126.7 | 0.778 | 2.854 | 2.076 | 0.1 |
| *Sphingopyxis* | 411 | 1.5 | 7439 | 126.7 | 2.613 | 3.871 | 1.257 | 0.9 |
| *Afipia* | 146 | 21.6 | 2001 | 209.3 | 2.164 | 3.301 | 1.136 | 1.0 |
| *Phenylobacterium* | 172 | 19.8 | 562 | 114.4 | 2.235 | 2.749 | 0.514 | 0.6 |
| *Methylopila* | 13 | 1.9 | 36 | 10.6 | 1.113 | 1.556 | 0.442 | |
| *Streptomyces* | 177 | 23.3 | 1034 | 151.9 | 2.247 | 3.014 | 0.766 | |
| *Reyranella* | 3 | 0.8 | 1 | 0.0 | 0.477 | 0.000 | -0.477 | 0.4 |
| *Rhodopseudomonas* | 0 | 0.0 | 0 | 0.0 | NA | NA | NA | 0.0 |
| Unidentified (Alcaligenaceae) | 13201 | 0.7 | 87 | 6.5 | 4.120 | 1.939 | -2.181 | 0.9 |
| *Pseudomonas* | 31674 | 1524.5 | 586 | 114.6 | 4.500 | 2.767 | -1.732 | 0.6 |
| Bray-curtis distance | 0.712 | NA | 0.848 | NA | NA | NA | 1.026 | 1.0 |
| 16S Copy number / ng DNA | 2.46E+09 | 211.5 | 3.40E+09 | 13.5 | 0.139 | 9.531 | -1.146 | 0.0 |
| **Archaea** | | | | | | | | |
| Number of reads | 14048 | 6561.0 | 11757 | 103.8 | 4.147 | 4.070 | -0.077 | 0.0 |
| 16S Copy number / ng DNA | 5.35E+08 | 18.4 | 8.73E+07 | 109.4 | 8.728 | 6.224 | -4.608 | 0.0 |
| **Fungi** | | | | | | | | |
| *Chrysosporium* | 135 | 42.7 | 5763 | 29.6 | 2.130 | 3.760 | 1.630 | |
| *Pseudogymnoascus* | 6731 | 3.9 | 103 | 51.4 | 3.828 | 2.012 | -1.815 | 0.7 |
| *Illyonectria* | 42 | 5.1 | 87 | 58.4 | 1.623 | 1.939 | 0.316 | |
| *Trichoderma* | 3313 | 729.8 | 144 | 37.9 | 3.520 | 2.158 | -1.361 | 0.6 |
| *Serendipita* | 0 | 0.0 | 0 | 0.0 | NA | NA | NA | 0.0 |
| *Cephalotrichum* | 298 | 70.7 | 711 | 73.3 | 2.474 | 2.851 | 0.377 | |
| Unidentified (Hypocreales) | 3882 | 101.9 | 161 | 22.7 | 3.589 | 2.206 | -1.382 | 0.9 |
| *Oidodendron* | 6 | 2.0 | 1 | 0.0 | 0.778 | 0.000 | -0.778 | 0.6 |
| Unidentified | 400 | 72.2 | 1777 | 92.5 | 2.602 | 3.249 | 0.647 | |
| *Clonostachys* | 4127 | 447.7 | 3 | 0.0 | 3.615 | 0.477 | -3.138 | 0.0 |
| *Gliomastix* | 78 | 9.6 | 332 | 11.7 | 1.892 | 2.521 | 0.629 | |
| *Ochroconis* | 267 | 1.1 | 1248 | 64.0 | 2.426 | 3.096 | 0.669 | 0.7 |
| *Mortierella* | 70 | 6.8 | 298 | 65.0 | 1.845 | 2.474 | 0.629 | 0.3 |
| *Saccharomyces* | 7 | 2.4 | 0 | 0.0 | 0.845 | NA | NA | 0.0 |
| Bray-curtis distance | 0.762 | NA | 0.733 | NA | NA | NA | -0.037 | |
| ITS Copy number / ng DNA | 5.44E+08 | 91.9 | 2.89E+09 | 101.9 | 8.735 | 9.457 | -1.214 | 0.5 |

| | Raw data: Unmarked surface | Raw data: Unmarked surfaces (SD) | Raw data: Dark zone |
|---|---|---|---|
| **Bacteria** | | | |
| *Niabella* | 0 | 0.0 | 0 |
| *Steroidobacter* | 0 | 0.0 | 0 |
| *Acidothermus* | 0 | 0.0 | 0 |
| *Phaselicystis* | 29 | 11.8 | 1029 |
| *Saccharopolyspora* | 20 | 5.6 | 24 |
| *Nitrospira* | 15 | 1.2 | 2 |
| *Jatrophihabitans* | 167 | 27.1 | 8 |
| *Nonomuraea* | 60 | 20.7 | 114 |
| *Tundrisphaera* | 2 | 0.4 | 0 |
| *Bryobacter* | 160 | 62.3 | 266 |
| *Flavihumibacter* | 0 | 0.0 | 0 |
| *Actinophytocola* | 104 | 39.0 | 249 |
| *Alysiosphaera* | 0 | 0.0 | 16 |
| *Galbitalea* | 0 | 0.0 | 0 |
| *Microlunatus* | 5 | 0.5 | 9 |
| Unidentified (Solirubrobactera | 8 | 0.8 | 3 |
| Unidentified (Thermomicrobia | 58 | 0.0 | 174 |
| *Jiangella* | 95 | 35.8 | 2783 |
| *Sphingopyxis* | 1685 | 5.6 | 6916 |
| *Afipia* | 229 | 70.1 | 5435 |
| *Phenylobacterium* | 70 | 9.6 | 139 |
| *Methylopila* | 244 | 65.2 | 7 |
| *Streptomyces* | 104 | 8.7 | 188 |
| *Reyranella* | 1 | 0.0 | 1 |
| *Rhodopseudomonas* | 0 | 0.0 | 0 |
| Unidentified (Alcaligenaceae) | 6622 | 39.0 | 15 |
| *Pseudomonas* | 18412 | 1867.6 | 171 |
| Bray-curtis distance | 0.556 | NA | 0.733 |
| 16S Copy number / ng DNA | 2.56E+10 | 119.4 | 2.44E+09 |
| **Archaea** | | | |
| Number of reads | 12375 | 484.6 | 244 |
| 16S Copy number / ng DNA | 2.64E+10 | 127.9 | 3.30E+07 |
| **Fungi** | | | |
| *Chrysosporium* | 340 | 11.0 | 1312 |
| *Pseudogymnoascus* | 3876 | 9.6 | 17 |
| *Illyonectria* | 78 | 21.6 | 8 |
| *Trichoderma* | 955 | 10.3 | 287 |
| *Serendipita* | 4 | 0.0 | 0 |
| *Cephalotrichum* | 465 | 104.5 | 70 |
| Unidentified (Hypocreales) | 1369 | 132.5 | 32 |
| *Oidodendron* | 11 | 4.4 | 0 |
| Unidentified | 1008 | 52.7 | 463 |
| *Clonostachys* | 866 | 91.4 | 0 |
| *Gliomastix* | 450 | 75.2 | 32 |
| *Ochroconis* | 562 | 4.8 | 6243 |
| *Mortierella* | 2285 | 835.4 | 181 |
| *Saccharomyces* | 128 | 6.4 | 218 |
| Bray-curtis distance | 0.668 | NA | 0.751 |
| ITS Copy number / ng DNA | 2.63E+09 | 5112.9 | 3.44E+09 |

| Passage vertical | | | | | Bulls center | | | | | | | | Bulls left | | | | | | | | Bulls wall | | | | | | | | Airlock-2 wall | | | | | | | |
|---|---|---|---|---|---|---|---|---|---|---|---|---|---|---|---|---|---|---|---|---|---|---|---|---|---|---|---|---|---|---|---|---|---|---|---|---|
| | Log data | | | | Raw data | | | | Log data | | | | Raw data | | | | Log data | | | | Raw data | | | | Log data | | | | Raw data | | | | Log data | | | |
| Dark zone (SD) | Unmarked surface (log) | Dark zone (log) | Delta log | **Score** | Unmarked surface | Unmarked surfaces (SD) | Dark zone | Dark zone (SD) | Unmarked surface (log) | Dark zone (log) | Delta log | **Score** | Unmarked surface | Unmarked surfaces (SD) | Dark zone | Dark zone (SD) | Unmarked surface (log) | Dark zone (log) | Delta log | **Score** | Unmarked surface | Unmarked surfaces (SD) | Dark zone | Dark zone (SD) | Unmarked surface (log) | Dark zone (log) | Delta log | **Score** | Unmarked surface | Unmarked surfaces (SD) | Dark zone | Dark zone (SD) | Unmarked surface (log) | Dark zone (log) | Delta log | **Score** |
| 0.0 | NA | NA | NA | **0.0** | 0 | 0.0 | 0 | 0.0 | NA | NA | NA | **0.0** | 0 | 0.0 | 0 | 0.0 | NA | NA | NA | **0.0** | 0 | 0.0 | 0 | 0.0 | NA | NA | NA | **0.0** | 0 | 0.0 | 0 | 0.0 | 0.000 | 0.000 | NA | **0.0** |
| 0.0 | NA | NA | NA | **0.0** | 0 | 0.0 | 0 | 0.0 | NA | NA | NA | **0.0** | 2 | 0.5 | 0 | 0.0 | 0.301 | NA | NA | **0.0** | 1 | 0.0 | 0 | 0.0 | 0.000 | NA | NA | **0.0** | 0 | 0.0 | 0 | 0.0 | 0.000 | 0.000 | NA | **0.0** |
| 0.8 | NA | NA | NA | **0.0** | 3 | 0.8 | 1 | 0.0 | 0.477 | 0.000 | -0.477 | | 0 | 0.0 | 0 | 0.0 | NA | NA | NA | **0.0** | 0 | 0.0 | 0 | 0.0 | NA | NA | NA | **0.0** | 1 | 0.0 | 0 | 0.0 | 0.000 | 0.000 | NA | **0.0** |
| 85.4 | 1.462 | 3.012 | 1.550 | **0.7** | 11 | 2.0 | 160 | 28.5 | 1.041 | 2.204 | 1.162 | **0.6** | 111 | 31.2 | 545 | 75.0 | 2.045 | 2.736 | 0.691 | **0.5** | 728 | 45.2 | 16 | 4.2 | 2.862 | 1.204 | -1.658 | | 150 | 55.9 | 18 | 4.9 | 2.176 | 1.255 | -0.920 | |
| 0.5 | 1.301 | 1.380 | 0.079 | **0.0** | 64 | 1.2 | 17 | 0.5 | 1.806 | 1.230 | -0.575 | | 168 | 1.2 | 36 | 0.0 | 2.225 | 1.556 | -0.669 | | 405 | 4.2 | 3 | 0.0 | 2.607 | 0.477 | -2.130 | | 33 | 3.6 | 13 | 0.4 | 1.519 | 1.113 | -0.404 | |
| 0.8 | 1.176 | 0.301 | -0.875 | | 5 | 0.8 | 27 | 1.0 | 0.698 | 1.431 | 0.732 | **0.5** | 39 | 11.3 | 35 | 14.2 | 1.591 | 1.544 | -0.046 | | 1617 | 80.7 | 126 | 48.9 | 3.208 | 2.100 | -1.108 | | 351 | 21.7 | 703 | 142.8 | 2.545 | 2.846 | 0.301 | **0.0** |
| 1.0 | 2.222 | 0.903 | -1.319 | | 42 | 8.2 | 9 | 1.3 | 1.623 | 0.954 | -0.669 | | 802 | 55.9 | 65 | 18.2 | 2.904 | 1.812 | -1.091 | | 1471 | 21.6 | 160 | 48.1 | 3.167 | 2.204 | -0.963 | | 116 | 1.7 | 342 | 42.8 | 2.064 | 2.534 | 0.469 | **0.4** |
| 22.7 | 1.778 | 2.056 | 0.278 | **0.0** | 27 | 2.7 | 74 | 11.3 | 1.431 | 1.869 | 0.437 | **0.3** | 35 | 10.1 | 11 | 0.7 | 1.544 | 1.041 | -0.502 | | 1656 | 66.2 | 4 | 0.8 | 3.219 | 0.602 | -2.617 | | 194 | 27.1 | 40 | 6.1 | 2.287 | 1.602 | -0.685 | |
| 0.0 | 0.301 | | NA | **0.0** | 10 | 0.0 | 56 | 0.0 | 1.000 | 1.748 | 0.748 | **0.6** | 14 | 0.8 | 156 | 0.0 | 1.146 | 2.193 | 1.046 | **0.8** | 1316 | 0.4 | 261 | 0.4 | 3.119 | 2.416 | -0.702 | | 240 | 3.9 | 577 | 0.4 | 2.380 | 2.761 | 0.380 | **0.0** |
| 16.9 | 2.204 | 2.424 | 0.220 | **0.1** | 106 | 16.7 | 1242 | 147.4 | 2.025 | 3.094 | 1.068 | **0.8** | 534 | 96.5 | 1162 | 194.1 | 2.727 | 3.065 | 0.337 | **0.3** | 818 | 75.2 | 629 | 194.1 | 2.912 | 2.798 | -0.114 | | 282 | 8.8 | 496 | 42.2 | 2.450 | 2.695 | 0.245 | **0.0** |
| 0.0 | NA | NA | NA | **0.0** | 1 | 0.4 | 28 | 8.6 | 0.000 | 1.447 | 1.447 | **1.0** | 0 | 0.0 | 0 | 0.0 | NA | NA | NA | **0.0** | 0 | 0.0 | 0 | 0.0 | 0.000 | 0.000 | 0.000 | **0.0** | 0 | 0.0 | 51 | 10.4 | 0.000 | 1.707 | NA | **0.0** |
| 24.3 | 2.017 | 2.396 | 0.379 | **0.4** | 98 | 2.3 | 2 | 0.0 | 1.991 | 0.301 | -1.690 | | 86 | 4.0 | 480 | 126.3 | 1.934 | 2.681 | 0.746 | **0.6** | 2170 | 38.7 | 1156 | 318.5 | 3.336 | 3.062 | -0.273 | | 210 | 42.9 | 2366 | 235.2 | 2.322 | 3.374 | 1.051 | **0.8** |
| 0.0 | NA | 1.204 | NA | **0.0** | 2 | 0.8 | 11 | 0.0 | 0.301 | 1.041 | 0.740 | **0.6** | 40 | 2.1 | 319 | 2.5 | 1.602 | 2.503 | 0.901 | **0.8** | 27 | 0.4 | 29 | 0.0 | 1.431 | 1.462 | 0.031 | **0.0** | 12 | 2.0 | 227 | 1.9 | 1.079 | 2.356 | 1.276 | **0.9** |
| 0.0 | NA | NA | NA | **0.0** | 0 | 0.0 | 0 | 0.0 | NA | NA | NA | **0.0** | 0 | 0.0 | 0 | 0.0 | NA | NA | NA | **0.0** | 1 | 0.0 | 16 | 6.0 | 0.000 | 1.204 | 1.204 | **1.0** | 6 | 0.4 | 80 | 5.6 | 0.778 | 1.903 | 1.124 | **0.9** |
| 0.3 | 0.698 | 0.954 | 0.255 | **0.0** | 14 | 1.6 | 8 | 2.8 | 1.146 | 0.903 | -0.243 | | 410 | 94.2 | 38 | 4.5 | 2.612 | 1.579 | -1.033 | | 233 | 3.7 | 98 | 9.6 | 2.367 | 1.991 | -0.376 | | 25 | 4.1 | 63 | 14.0 | 1.397 | 1.799 | 0.401 | **0.3** |
| 0.5 | 0.903 | 0.477 | -0.425 | | 38 | 2.2 | 6 | 2.4 | 1.579 | 0.778 | -0.801 | | 20 | 36.0 | 11 | 8.0 | 1.301 | 1.041 | -0.259 | | 649 | 7.6 | 95 | 2.0 | 2.812 | 1.977 | -0.834 | | 453 | 55.8 | 25 | 6.0 | 2.656 | 1.397 | -1.258 | |
| 0.0 | 1.763 | 2.240 | 0.477 | **0.5** | 224 | 0.0 | 105 | 0.4 | 2.350 | 2.021 | -0.329 | | 707 | 0.0 | 192 | 0.0 | 2.849 | 2.283 | -0.566 | | 3638 | 0.0 | 1616 | 0.8 | 3.560 | 3.208 | -0.352 | | 1954 | 0.9 | 1657 | 19.3 | 3.290 | 3.219 | -0.071 | |
| 208.5 | 1.977 | 3.444 | 1.466 | **0.8** | 145 | 20.0 | 1818 | 407.8 | 2.161 | 3.259 | 1.098 | **1.0** | 437 | 66.4 | 1216 | 232.1 | 2.640 | 3.084 | 0.444 | **0.5** | 1787 | 185.3 | 214 | 41.7 | 3.252 | 2.330 | -0.921 | | 790 | 197.1 | 430 | 27.2 | 2.897 | 2.633 | -0.264 | |
| 8.5 | 3.226 | 3.839 | 0.613 | **0.6** | 602 | 20.0 | 17174 | 407.8 | 2.779 | 4.234 | 1.455 | **0.8** | 2025 | 66.4 | 11420 | 232.1 | 3.306 | 4.057 | 0.751 | **0.7** | 2239 | 185.3 | 28979 | 41.7 | 3.350 | 4.462 | 1.112 | **1.0** | 1824 | 197.1 | 6075 | 27.2 | 3.261 | 3.783 | 0.522 | **0.5** |
| 276.9 | 2.359 | 3.735 | 1.375 | **0.8** | 339 | 45.0 | 4699 | 607.6 | 2.530 | 3.670 | 1.141 | **1.0** | 137 | 23.1 | 2553 | 336.2 | 2.136 | 3.407 | 1.270 | **0.9** | 1820 | 212.6 | 6926 | 579.1 | 3.260 | 3.840 | 0.580 | **0.6** | 1398 | 162.3 | 6459 | 575.6 | 3.145 | 3.810 | 0.664 | **0.7** |
| 12.4 | 1.845 | 2.143 | 0.297 | **0.4** | 112 | 18.0 | 487 | 52.5 | 2.049 | 2.687 | 0.638 | **0.7** | 181 | 17.3 | 160 | 12.1 | 2.257 | 2.204 | -0.053 | | 65 | 29.5 | 180 | 31.0 | 1.812 | 2.255 | 0.442 | **0.5** | 111 | 11.1 | 199 | 31.7 | 2.045 | 2.298 | 0.253 | **0.3** |
| 1.1 | 2.387 | 0.845 | -1.542 | **0.8** | 998 | 87.0 | 19 | 4.9 | 2.999 | 1.278 | -1.720 | **0.6** | 195 | 31.8 | 76 | 25.6 | 2.290 | 1.880 | -0.409 | **0.5** | 1 | 0.0 | 118 | 31.2 | 0.000 | 2.071 | 2.071 | | 149 | 0.4 | 12 | 2.2 | 2.173 | 1.079 | -1.094 | **1.0** |
| 23.1 | 2.017 | 2.274 | 0.257 | | 439 | 46.8 | 290 | 82.1 | 2.642 | 2.462 | -0.180 | **0.1** | 583 | 140.1 | 106 | 25.3 | 2.765 | 2.025 | -0.740 | **0.7** | 580 | 87.2 | 2407 | 628.2 | 2.763 | 3.381 | 0.618 | **0.6** | 480 | 50.8 | 1490 | 128.2 | 2.681 | 3.173 | 0.491 | |
| 0.0 | 0.000 | 0.000 | 0.000 | **0.0** | 131 | 20.6 | 25 | 5.4 | 2.117 | 1.397 | -0.719 | **0.6** | 20 | 4.7 | 33 | 10.1 | 1.301 | 1.518 | 0.217 | | 402 | 3.4 | 52 | 20.3 | 2.604 | 1.716 | -0.888 | **0.7** | 32 | 4.5 | 134 | 5.9 | 1.505 | 2.127 | 0.621 | |
| 0.0 | 0.000 | 0.000 | 0.000 | **0.0** | 62 | 16.1 | 0 | 0.0 | 1.792 | NA | NA | **0.0** | 1 | 0.0 | 0 | 0.0 | 0.000 | NA | NA | **0.0** | 0 | 0.0 | 0 | 0.0 | NA | NA | NA | **0.0** | 132 | 0.0 | 0 | 0.0 | 0.000 | 0.000 | NA | **0.0** |
| 24.3 | 3.820 | 1.176 | -2.644 | **0.9** | 2703 | 2.3 | 270 | 0.4 | 3.431 | 2.431 | -1.000 | **0.5** | 1569 | 4.0 | 703 | 126.3 | 3.195 | 2.846 | -0.348 | **0.0** | 21 | 8.7 | 90 | 8.5 | 1.322 | 1.954 | 0.632 | | 7051 | 342.9 | 19 | 5.2 | 3.848 | 1.278 | -2.569 | **0.8** |
| 16.6 | 4.265 | 2.232 | -2.032 | **0.7** | 46213 | 790.6 | 396 | 79.2 | 4.664 | 2.597 | -2.067 | **0.7** | 15890 | 4418.1 | 225 | 33.6 | 4.201 | 2.352 | -1.848 | **0.6** | 14 | 6.0 | 50 | 12.2 | 1.146 | 1.698 | 0.552 | | 28456 | 2.0 | 10 | 1.2 | 4.454 | 1,000 | -3.454 | **0.9** |
| NA | NA | NA | -0.205 | **0.0** | 0.769 | NA | 0.781 | NA | NA | NA | -0.231 | **0.0** | 0.769 | NA | 0.781 | NA | NA | NA | 1.066 | **1.0** | 0.713 | NA | 0.869 | NA | NA | NA | 4.302 | **0.0** | 0.912 | NA | 0.863 | NA | NA | NA | -0.125 | **0.0** |
| 33.5 | -1.021 | 9.386 | 0.338 | **0.3** | 1.06E+09 | 272.7 | 2.73E+09 | 255.9 | 0.411 | 9.436 | 0.322 | **0.3** | 4.5E+09 | 94.8 | 6.12E+09 | 1202.2 | 0.133 | 9.786 | 1.661 | **0.6** | 2.79E+09 | 809.8 | 1.99E+08 | 140.1 | -1.146 | 8.299 | 0.139 | **0.0** | 1.93E+09 | 65.2 | 4.20E+09 | 80.4 | 0.338 | 9.623 | -1.021 | **0.0** |
| | | | | | | | | | | | | | | | | | | | | | | | | | | | | | | | | | | | | |
| 76.7 | 4.092 | 2.387 | -1.705 | **0.5** | 13455 | 699.3 | 1006 | 65.3 | 4.128 | 3.002 | -1.126 | **1.0** | 5736 | 3913.5 | 437 | 39.1 | 3.758 | 2.640 | -1.118 | **1.0** | 33226 | 16971.3 | 267 | 245.3 | 4.521 | 2.426 | -2.094 | **0.0** | 14926 | 2664.6 | 3928 | 201.9 | 4.173 | 3.594 | -0.579 | **0.6** |
| 1275.9 | 10.421 | 5.646 | -3.454 | **0.3** | 3.31E+08 | 34.8 | 4.72E+07 | 412.8 | 8.520 | 5.853 | -3.131 | **1.0** | 2.83E+09 | 793.3 | 8.18E+07 | 53.3 | 9.451 | 6.184 | -1.669 | **0.9** | 3.53E+09 | 448.4 | 8.65E+06 | 61.4 | 9.548 | 4.939 | -2.503 | **0.3** | 5.51E+09 | 86.6 | 9.64E+07 | 865.6 | 9.741 | 6.286 | -4.775 | **0.7** |
| | | | | | | | | | | | | | | | | | | | | | | | | | | | | | | | | | | | | |
| 80.4 | 2.531 | 3.117 | 0.586 | | 1207 | 66.2 | 24 | 6.4 | 3.081 | 1.380 | -1.701 | **0.6** | 14749 | 94.2 | 4151 | 653.0 | 4.168 | 3.618 | -0.550 | **0.0** | 50.4 | 6.1 | 5 | 2.8 | 1.702 | 0.698 | -1.003 | **0.3** | 135 | 37.9 | 4 | 1.8 | 2.130 | 0.602 | -1.528 | **0.5** |
| 2.6 | 3.588 | 1.230 | -2.357 | **0.8** | 1935 | 626.8 | 11 | 7.7 | 3.286 | 1.041 | -2.245 | **0.8** | 5397 | 81.0 | 18 | 8.7 | 3.732 | 1.255 | -2.476 | **0.8** | 514.8 | 86.7 | 9 | 2.5 | 2.711 | 0.954 | -1.757 | **0.6** | 32 | 6.1 | 17 | 3.7 | 1.505 | 1.230 | -0.274 | **0.0** |
| 6.7 | 1.892 | 0.903 | -0.989 | **0.4** | 26 | 2.0 | 1 | 0.0 | 1.414 | 0.000 | -1.414 | **0.6** | 35 | 4.7 | 5 | 0.2 | 1.544 | 0.698 | -0.845 | **0.4** | 117.6 | 45.7 | 564 | 183.7 | 2.070 | 2.751 | 0.680 | | 3 | 2.1 | 704 | 281.6 | 0.477 | 2.847 | 2.370 | |
| 3.0 | 2.980 | 2.457 | -0.522 | **0.3** | 441 | 95.6 | 0 | 0.0 | 2.644 | NA | NA | **0.0** | 626 | 132.8 | 78 | 15.2 | 2.796 | 1.892 | -0.904 | **0.4** | 12792 | 398.5 | 6872 | 519.7 | 4.106 | 3.837 | -0.269 | **0.0** | 24 | 3.1 | 967 | 90.2 | 1.380 | 2.985 | 1.605 | |
| 0.0 | 0.602 | 0.000 | NA | **0.0** | 0 | 0.0 | 0 | 0.0 | NA | NA | NA | **0.0** | 0 | 0.0 | 0 | 0.0 | NA | NA | NA | **0.0** | 0 | 0.0 | 0 | 0.0 | NA | NA | NA | **0.0** | 0 | 0.0 | 0 | 0.0 | 0.000 | 0.000 | NA | **0.0** |
| 18.8 | 2.667 | 1.845 | -0.822 | **0.5** | 169 | 18.2 | 302 | 21.8 | 2.227 | 2.480 | 0.252 | | 59 | 9.8 | 26 | 6.4 | 1.770 | 1.414 | -0.355 | **0.0** | 13.2 | 2.7 | 2 | 0.0 | 1.120 | 0.301 | -0.819 | **0.5** | 298 | .5581348741 | 0 | 0.0 | 2.474 | 0.000 | NA | **0.0** |
| 4.7 | 3.136 | 1.505 | -1.631 | **1.0** | 1256 | 811.3 | 502 | 81.2 | 3.098 | 2.700 | -0.398 | **0.3** | 281 | 19.0 | 16 | 2.9 | 2.448 | 1.204 | -1.244 | **0.8** | 362.4 | 32.5 | 195 | 59.4 | 2.559 | 2.290 | -0.269 | **0.0** | 186 | 21.3 | 414 | 0.0 | 2.269 | 2.617 | 0.347 | |
| 0.0 | 1.041 | 0.000 | NA | **0.0** | 0 | 0.0 | 0 | 0.0 | NA | NA | NA | **0.0** | 6 | 0.0 | 0 | 0.0 | 0.778 | NA | NA | **0.0** | 13.2 | 0.0 | 0 | 0.0 | 1.120 | NA | NA | **0.0** | 0 | 0.0 | 0 | 0.0 | 0.000 | 0.000 | NA | |
| 42.0 | 3.003 | 2.665 | -0.337 | **0.3** | 9347 | 1211.4 | 3304 | 84.4 | 3.970 | 3.519 | -0.451 | **0.4** | 2706 | 889.6 | 359 | 10.2 | 3.432 | 2.555 | -0.877 | **0.7** | 6174 | 222.5 | 2058 | 287.4 | 3.790 | 3.313 | -0.477 | **0.5** | 2689 | 116.5 | 407 | 154.2 | 3.429 | 2.609 | -0.819 | **0.7** |
| 0.0 | 2.937 | 0.000 | NA | **0.0** | 38 | 8.0 | 1 | 0.0 | 1.579 | 0.000 | -1.579 | **0.6** | 16 | 1.1 | 2 | 0.3 | 1.204 | 0.301 | -0.903 | **0.9** | 49.2 | 5.3 | 0 | 0.0 | 1.691 | NA | NA | **0.0** | 17 | 7.5 | 19 | 5.4 | 1.230 | 1.278 | 0.048 | |
| 9.9 | 2.653 | 1.505 | -1.148 | **0.0** | 153 | 41.2 | 85 | 30.1 | 2.184 | 1.929 | -0.255 | **0.6** | 318 | 65.1 | 130 | 58.4 | 2.502 | 2.113 | -0.388 | **0.9** | 1116 | 99.3 | 175 | 17.4 | 3.047 | 2.243 | -0.804 | **0.0** | 222 | 50.9 | 98 | 3.4 | 2.346 | 1.991 | -0.355 | **0.0** |
| 891.9 | 2.749 | 3.795 | 1.045 | **1.0** | 540 | 30.4 | 784 | 172.1 | 2.732 | 2.894 | 0.161 | **0.0** | 274 | 89.5 | 2198 | 196.7 | 2.437 | 3.342 | 0.904 | **0.9** | 512.4 | 16.6 | 3375 | 873.8 | 2.709 | 3.528 | 0.818 | **0.8** | 19 | 7.3 | 1784 | 428.4 | 1.278 | 3.251 | 1.972 | **0.4** |
| 20.8 | 3.358 | 2.257 | -1.101 | | 676 | 88.1 | 260 | 48.4 | 2.829 | 2.414 | -0.414 | | 946 | 12.8 | 770 | 334.8 | 2.975 | 2.886 | -0.089 | | 6126 | 222.4 | 5711 | 164.8 | 3.787 | 3.756 | -0.030 | | 974 | 212.4 | 567 | 40.7 | 2.988 | 2.753 | -0.234 | |
| 9.8 | 2.107 | 2.338 | 0.231 | **0.0** | 12 | 2.9 | 1 | 0.5 | 1.079 | 0.000 | -1.079 | | 3 | 0.8 | 0 | 0.0 | 0.477 | NA | NA | **0.0** | 7.2 | 0.8 | 0 | 0.0 | 0.857 | NA | NA | **0.0** | 84 | 20.9 | 1 | 0.0 | 1.924 | 0.000 | -1.924 | |
| NA | NA | NA | 0.122 | **0.0** | 0.837 | NA | 0.89 | NA | NA | NA | 0.063 | **0.0** | 0.704 | NA | 0.847 | NA | NA | NA | 0.203 | **0.0** | 0.755 | NA | 0.848 | NA | NA | NA | 0.123 | **0.0** | 0.847 | NA | 0.736 | NA | NA | NA | -0.137 | **0.0** |
| 12.9 | 9.420 | 9.536 | 0.350 | **0.0** | 4.64E+08 | 124.8 | 1.21E+09 | 44.8 | 8.666 | 9.082 | 0.204 | **0.3** | 5.19E+09 | 216.3 | 6.64E+09 | 612.3 | 9.712 | 9.821 | 1.947 | **0.0** | 1.95E+09 | 18.4 | 1.19E+08 | 501.4 | 9.290 | 8.076 | 0.721 | **0.0** | 1.59E+09 | 74.6 | 3.57E+09 | 862.6 | 9.201 | 9.552 | 0.115 | **0.3** |
